# Interplay of Bridge Topology and Conformation in Phenylene‐Bridged Trityl Diradicals

**DOI:** 10.1002/chem.71384

**Published:** 2026-07-09

**Authors:** Kevin Lars Kopp, Tobias Hett, Andrea Pellegrini, Stefan Grimme, Olav Schiemann

**Affiliations:** ^1^ Clausius‐Institute for Physical and Theoretical Chemistry University of Bonn Bonn Germany; ^2^ Department of Industrial Chemistry “Toso Montanari” University of Bologna Bologna Italy; ^3^ Mulliken Center Clausius‐Institute for Physical and Theoretical Chemistry University of Bonn Bonn Germany

**Keywords:** EPR, exchange coupling, magnetometry, molecular magnets, trityl

## Abstract

Tuning the exchange coupling constant *J* is a valuable tool in the design of molecular systems for applications in molecular magnetism and quantum information science. The phenylene group is an archetypal bridge to influence the sign and magnitude of *J* via topology and substitution. Motivated by this, we synthesized an *m*‐phenylene‐bridged trityl diradical (***m*‐ben‐(TAM)_2_^••^**) and explored its magnetic coupling in comparison to the *p*‐phenylene‐ and *p*‐xylene‐bridged analogues. We find a weak antiferromagnetic coupling of −4 GHz for ***m*‐ben‐(TAM)_2_^••^** in the powder, which increases to −169 GHz in solution. The antiferromagnetic nature of the spin‐spin coupling is rationalized by the large dihedral angle between the trityl and bridge aryl groups, which diminishes the ferromagnetic π‐contribution. The increase in the magnitude of *J* by going from the powder to the solution state is rationalized by a large structural change, as investigated by density functional theory (DFT) calculations and molecular dynamics (MD) simulations. We speculate that these geometric changes lead to changes in the intramolecular through‐bond and through‐space contributions to *J*, but can't rule out that it is also connected to intermolecular contributions in the powder. These results highlight that the design of ferromagnetic coupling cannot be based only on the topological rule.

## Graphical abstract

We present a *meta*‐phenylene‐bridged trityl diradical that exhibits an unexpectedly weak antiferromagnetic exchange coupling *J* with a strong environment‐dependent magnitude. Combined VSM, EPR, DFT, and MD studies reveal that structural flexibility modulates σ‐, π‐, and through‐space exchange contributions, highlighting the crucial role of geometry in the sign and magnitude of *J*.

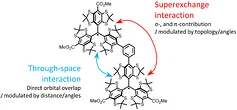

## 1 Introduction

Molecular, exchange‐coupled multi‐spin systems are of interest in the field of molecular magnetism [1], quantum information science (QIS) [2], and dynamic nuclear polarization (DNP) [3]. Such systems can be synthesized by connecting spin‐bearing groups via molecular bridges. These bridges can mediate the magnetic exchange interaction *J* via a super exchange mechanism that involves both, the σ‐ and the π‐bonds [4]. The mediating properties of the π‐system depend on the overlap between the π‐orbitals and can be tuned by synthetic alteration of the spin‐bearing group or the bridge. An archetypal bridge is phenylene, whose mediating properties can be tuned via, for example, the topology, that is, attachment of the spin centers in *ortho*‐, *meta*‐, and *para*‐positions relative to each other [5, 6, 7, 8, 9, 10]. Based purely on topology, a *p*‐phenylene connection should lead to antiferromagnetic coupling, whereas *m*‐phenylene yields ferromagnetic coupling [11, 12, 13, 14]. Simultaneously, substituents on the bridge can alter the electronic and geometric properties of the bridge and thereby also influence the sign and magnitude of *J*. It is, thus, of interest to study the interplay of topology and substitution for a given spin system. For example, Kanzaki et al. [15] and Hattori et al. [16] found for *p*‐phenylene‐bridged bis(nitronyl nitroxide) **1**–**3** and bis(3,5‐dichloro‐4‐pyridyl)bis(2,4,6‐trichlorophenyl)methyl radicals **4** and **5** antiferromagnetic coupling, as expected for this topology. In addition, they observed a decrease in the magnitude of *J* upon substitution of the bridge, which was related to the increased dihedral angle *θ* between the radical group and the phenylene bridge.

For the dependence of *J* on *θ*, Equation (1) has been derived [4, 6]

(1) $$J=J_{\pi }\;\cdot \cos^{2}\theta +J_{\sigma }.$$

Here, *J_π_* describes the π‐contribution to the exchange interaction and *J_σ_* the σ‐contribution. In contrast to the topology rule, studies by Stasiw et al. [4] and Shultz et al. [6] on a series of mixed nitronyl nitroxide/semiquinone diradicals **6**–**12** with *p*‐phenylene bridges revealed ferromagnetic coupling in all cases. Substitution of the bridge led in each case to an increase in *θ* and again a decrease in the magnitude of *J*.

Based on the topology rule, the *m*‐phenylene topology leads to ferromagnetic coupling, as shown on a plethora of systems, including, for example, nitroxide [17], mixed nitronyl nitroxide/verdazyl [18], and perchlorinated trityl diradicals [19]. However, there are also several examples that show that this is not always the case (Figure 1B) [20, 21, 22, 23]. For example, Terada et al. studied *m*‐ and *p*‐phenylene‐bridged bis(10‐phenyl‐5,10‐dihydrophenazine) dictations and observed strong antiferromagnetic couplings in both systems [20]. The same was found by Yamamoto et al. for *p*‐ and *m*‐phenylene‐bridged porphyrin *meso*‐oxy radical dimers [21]. Bodzioch et al. studied *para*‐ and *meta*‐phenylene‐bridged verdazyl diradicals, connected either via the nitrogen or the carbon atom of the verdazyl moiety, and also observed antiferromagnetic interactions in each case [22]. These observations were rationalized by the large *θ* in these systems (between 70° and 90°), which minimizes *J_π_*. Thus, *J* is in all these systems dominated by *J_σ_*, which is antiferromagnetic in nature. This has been extensively underpinned by computational methods [14, 24, 25].

**FIGURE 1:**
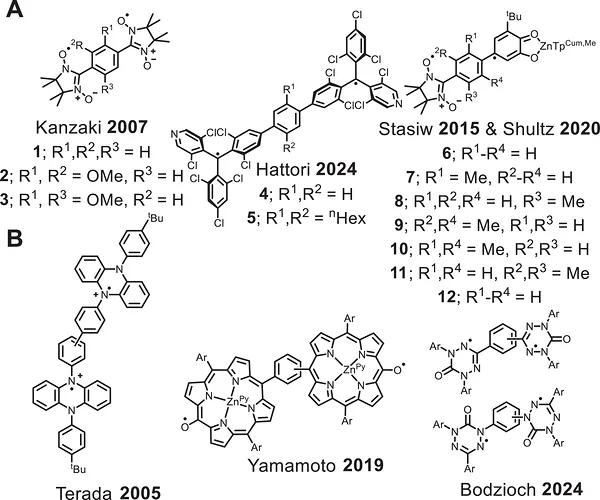
Examples for *J*‐coupled diradicals. (A) Diradicals for which the dependence of *J* on the dihedral angle and (B) on the topology was studied.

Here, we want to study the interplay of topology and substitution on the example of the tetrathiatriarylmethyl radicals (TAM‐type trityl radicals). In TAM radicals, roughly 70% of the spin density resides on the central carbon atom and 30% is delocalized over the three aryl units [26, 27]. They exhibit narrow linewidth and long electron‐spin coherence times in electron paramagnetic resonance (EPR) experiments [28, 29, 30]. Furthermore, C─C cross‐coupling reactions with trityl radicals have been established that facilitated the synthesis of π‐conjugated trityl‐diradical and trityl radical‐chromophore systems [31, 32, 33, 34]. Recently, the synthesis of a series of oligo(*p*‐phenylene)‐bridged TAM diradicals has been published [33], amongst which were the *p*‐phenylene‐ and *p*‐xylene‐bridged bistrityl diradicals. In this latter case, the substitution of the phenylene group with two methyl groups (*p*‐xylene bridge) led to a large decrease in the magnitude of the antiferromagnetic coupling, that is, from −235 to −82 GHz, respectively. In contrast, TAM coupled via a *p*‐phenylene‐bridge to a Cu(II)porphyrin led to a weak ferromagnetic coupling [35], while two *m*‐phenylene‐bridged tris(2,4,6‐trichlorophenyl)methyl (TTM) radicals gave rise to ferromagnetic coupling based on computational methods [36]. Similarly, ferromagnetic coupling was found experimentally for a perchlorinated bistrityl sharing one of its phenyls in *meta*‐connectivity [19]. Thus, it is not easy to predict the magnitude and sign of *J* for such trityl diradicals. We, therefore, synthesized the *m*‐phenylene bridged trityl diradical ***m*‐ben‐(TAM)_2_^••^** and compared it to the previously published systems ***p*‐ben‐(TAM)_2_^••^** and ***p*‐xyl‐(TAM)_2_^••^** (Figure 2). Aside from determining *J* of ***m*‐ben‐(TAM)_2_^••^** in frozen solution via X‐band EPR (9.5 GHz) experiments, we also investigate all three molecules via vibrating sample magnetometry (VSM) in the powder, and use molecular dynamics (MD) simulations and density functional theory (DFT) calculations to rationalize the experimental observations.

**FIGURE 2:**
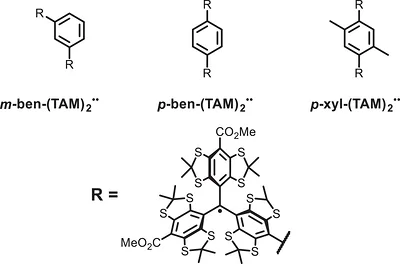
Molecular structures of the trityl diradicals studied in this work.

## 2 Results and Discussion

### 2.1 Synthesis

We synthesized ***m*‐ben‐(TAM)_2_^••^** according to the synthesis of ***p*‐ben‐(TAM)_2_^••^** and ***p*‐xyl‐(TAM)_2_^••^** as shown in Scheme 1 [33, 37]. The iodinated trityl alcohol **13** was synthesized according to Chen et al. [38] and cross‐coupled via a Suzuki–Miyaura reaction with 1,3‐bis(4,4,5,5‐tetramethyl‐1,3,2‐dioxaborolan‐2‐yl)‐benzene to yield **14**. The bistritylalcohol **14** was obtained in a yield of 79%, similar to ***p*‐ben‐(TAM)_2_^••^** and ***p*‐xyl‐(TAM)_2_^••^**. Subsequent conversion to the diradical was achieved by reaction with triflic acid followed by reduction of the resulting cation with tin(II)chloride at a yield of 62% [39]. ***m*‐ben‐(TAM)_2_^••^** was characterized by high‐resolution mass spectrometry and MALDI, and its purity of at least 95% was confirmed via medium‐pressure liquid chromatography (MPLC) and NMR spectra of precursor **14** (Chapters S2 and S3). Notably, we also tried to synthesize the *ortho*‐substituted bistrityl diradical. However, using 1,2‐bis(4,4,5,5‐tetramethyl‐1,3,2‐dioxaborolan‐2‐yl)‐benzene for the cross‐coupling reaction to the bistritylalcohol did not lead to the expected compound, most likely due to high steric congestion.

**SCHEME 1:**
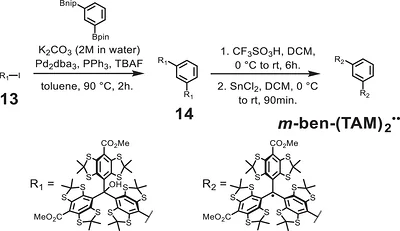
Synthesis of *m*‐ben‐(TAM)_2_^••^.

### 2.2 CW EPR

We assessed the magnitude of *J* for ***m*‐ben‐(TAM)_2_^••^** by recording its cw X‐Band (9.5 GHz) EPR spectrum in liquid toluene‐d_8_ (Figure 3A). We simulated the EPR spectrum using EasySpin [40], assuming two S = 1/2 systems, each with its own set of ^13^C hyperfine coupling constants *A*(^13^C), and included the spin exchange Hamiltonian H_ex_. As reported for similar trityl systems [27, 31, 32, 33], the spectrum consists of an intense central line, which originates from trityl molecules bearing only ^12^C carbon atoms or ^13^C carbon atoms with negligible hyperfine interaction, and a set of low‐intensity doublets caused by trityls carrying one ^13^C carbon atom at either the central (C_c_), ipso (C_i_), ortho (C_o_), para (C_p_), or meta (C_m_) position. The spectrum of ***m*‐ben‐(TAM)_2_^••^** could be simulated with the same set of parameters as previously published for ***p*‐ben‐(TAM)_2_^••^** and ***p*‐xyl‐(TAM)_2_^••^**, that is, an isotropic *g*‐value of 2.0031, the hyperfine coupling constants *A*(^13^C_c_) = 65.1 MHz, *A*(^13^ C_i_) = 31.2 MHz, and *A*(^13^C_o_) = 24.9 MHz [33], and a |*J*| > 500 MHz (the sign of *J* cannot be determined from simulations of liquid state spectra). As a result of the strong *J*‐coupling, the observable ^13^C hyperfine splittings are half of the ^13^C hyperfine splitting constants [31]. As for the literature‐known compounds, the doublets of the *para*‐ and *meta*‐carbon atoms, *A*(^13^C_p_) and *A*(^13^C_o_), could not be resolved because of the linewidth of the central line. We then determined *J* for ***m*‐ben‐(TAM)_2_^••^** in frozen solution from the temperature‐dependent signal intensity of the half‐field EPR transition ($\Delta m_{s}=-2$) at *g* = 4. We plotted the product of the background‐corrected and doubly integrated cw EPR signal intensity, *I*_DI_, and the temperature, *T*, against *T* and fitted the data using the Bleaney–Bowers equation (Equation 2) (Figure 3B) [41]

(2) $$I_{DI}T=C_{EPR}\left(\frac{1}{3+e^{-\frac{2J}{k_{B}T}}}\right)$$

where *C_EPR_* is a spectrometer‐specific constant, and *k_B_* is Boltzmann's constant. Performing the measurements in duplicates (Chapter S4.4), we obtained a mean value for *J* of −169(34) GHz (Figure 3B). Thus, the *m*‐phenylene bridge gives rise to an antiferromagnetic coupling with magnitude between that of ***p*‐ben‐(TAM)_2_^••^** and ***p*‐xyl‐(TAM)_2_^••^** (Table 1). We then fitted the frozen solution spectrum of ***m*‐ben‐(TAM)_2_^••^** in the *g* = 2 region ($\Delta m_{s}=1)$ assuming a triplet system (S = 1), an isotropic *g*‐value, and a zero‐field splitting parameter *D* (Chapter S4.2) to gain information about its geometric structure (Figure 3C). The inter‐spin distance *r*_EPR_ has been calculated from the zero‐field splitting constant *D* using Equation (3) [42]:

(3) $$D=\frac{3}{2}\;\frac{\mu_{0}g^{2}\mu_{B}^{2}}{4\pi \hbar^{2}}\frac{1}{r_{EPR}^{3}}$$

where *μ_0_* is the vacuum susceptibility, *ħ* the reduced Planck constant, and *μ_B_* Bohr's magneton. In Table 1, all electron spin–spin coupling parameters are summarized and compared to the published parameters for ***p*‐ben‐(TAM)_2_^••^** and ***p*‐xyl‐(TAM)_2_^••^**. As expected, *r_EPR_* is shorter in ***m*‐ben‐(TAM)_2_^••^** than in ***p*‐xyl‐(TAM)_2_^••^**, that is, 11.7 versus 12.7 Å. The surprisingly short *r_EPR_* of 11.8 Å found previously for ***p*‐ben‐(TAM)_2_^••^** has been attributed to its strong electron–electron spin–spin coupling, causing the point‐dipole approximation to fail [33].

**FIGURE 3:**
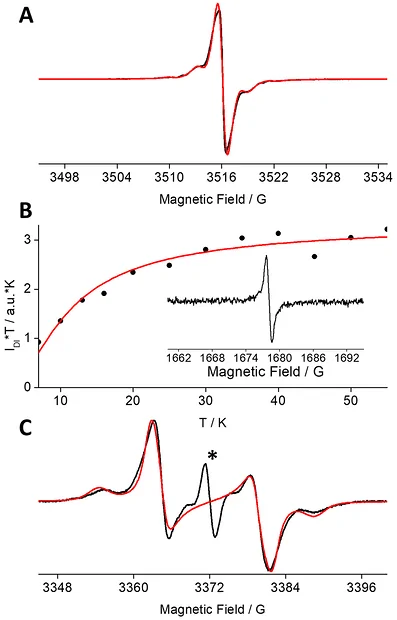
CW X‐Band EPR data of ***m*‐ben‐(TAM)_2_^••^** 50 µM in toluene‐d_8_. (A) The EPR spectrum (9.86 GHz) at room temperature. (B) The plot of the half‐field signal intensities I_DI_ multiplied by the respective temperature T in dependence on the temperature T for one of the duplicate measurements (*J* = −6.77 ± 0.45 cm^−1^). The inset shows the signal at half‐field at 7 K. (C) The spectrum (9.46 GHz) at 100 K. Experimental data are shown in black and the fits are overlaid in red, in each case. The asterisk denotes the position of the double quantum transition ($\Delta m_{s}=2$).

**TABLE 1:** The parameters obtained from the fitting of the cw X‐band EPR spectra and the VSM data of ***p*‐xyl‐(TAM)_2_^••^**, ***m*‐ben‐(TAM)_2_^••^**, and ***p*‐ben‐(TAM)_2_^••^**. The *J*‐values are based on the spin Hamiltonian H_ex_ = −2*J*S_1_S_2_.

Comp.	*m*‐ben‐(TAM)_2_^••^	*p*‐ben‐(TAM)_2_^••^	*p*‐xyl‐(TAM)_2_^••^
D/MHz	48.1(1)	47.8(1)	34.7(1)
*r*_EPR_/Å	11.7(1)	11.8(1)	12.7(1)
*J*_EPR_	−169(34) GHz = −6.5(11) cm^−1^	−235(17) GHz = −7.8(6) cm^−1^	−82(9) GHz = −2.7(3) cm^−1^
*J*_VSM_	−4(1) GHz = −0,13(3) cm^−1^	−254(1) GHz = −8.47(3) cm^−1^	−23(1) GHz = −0.77(3) cm^−1^
*C*/*cm^3^mol^−1^*	0.52(1)	0.47(1)	0.54(4)
*χ*_0_/_*_ 10^−4^ *cm^3^mol^−1^*	6.9(1)	6.0(1)	20.2(1)

### 2.3 Magnetometry

As a complementary method to EPR, vibrating sample magnetometry (VSM) in the temperature range of 1.8–300 K at an applied magnetic field of 10,000 Oe was used to determine *J* for all three compounds in the powder state. Measurements of the susceptibilities at a lower magnetic field led to a signal‐to‐noise ratio too low to analyze the data. The measured susceptibilities *χ_mol_* were multiplied by the temperature *T* and are shown as a function of *T* in Figure 4. The plots for both ***m*‐ben‐(TAM)_2_^••^** and ***p*‐xyl‐(TAM)_2_^••^** change their linear behavior only at very low temperature, indicating weak interaction between the spin centers (Figure 4A/C). Thus, *J* will be on a similar order of magnitude as the Zeeman splitting, which requires that the magnetic field *B* is included in the description of the temperature dependence of *χ_mol_* and thus, the use of Equation 4 [43]

(4) χmolT=CTBZ−g·eJJ22−gμBBkT+g·eJJ22+gμBBkT+χ0T,

where *C* is a constant prefactor, *χ_0_* is the temperature‐independent susceptibility [44], and *Z* is the partition function of the spin system, defined by Equation (5)

(5) Z=eJJ22−gμBBkT+eJJ22kT+eJJ22+gμBBkT+e−3J3J22kT.

**FIGURE 4:**
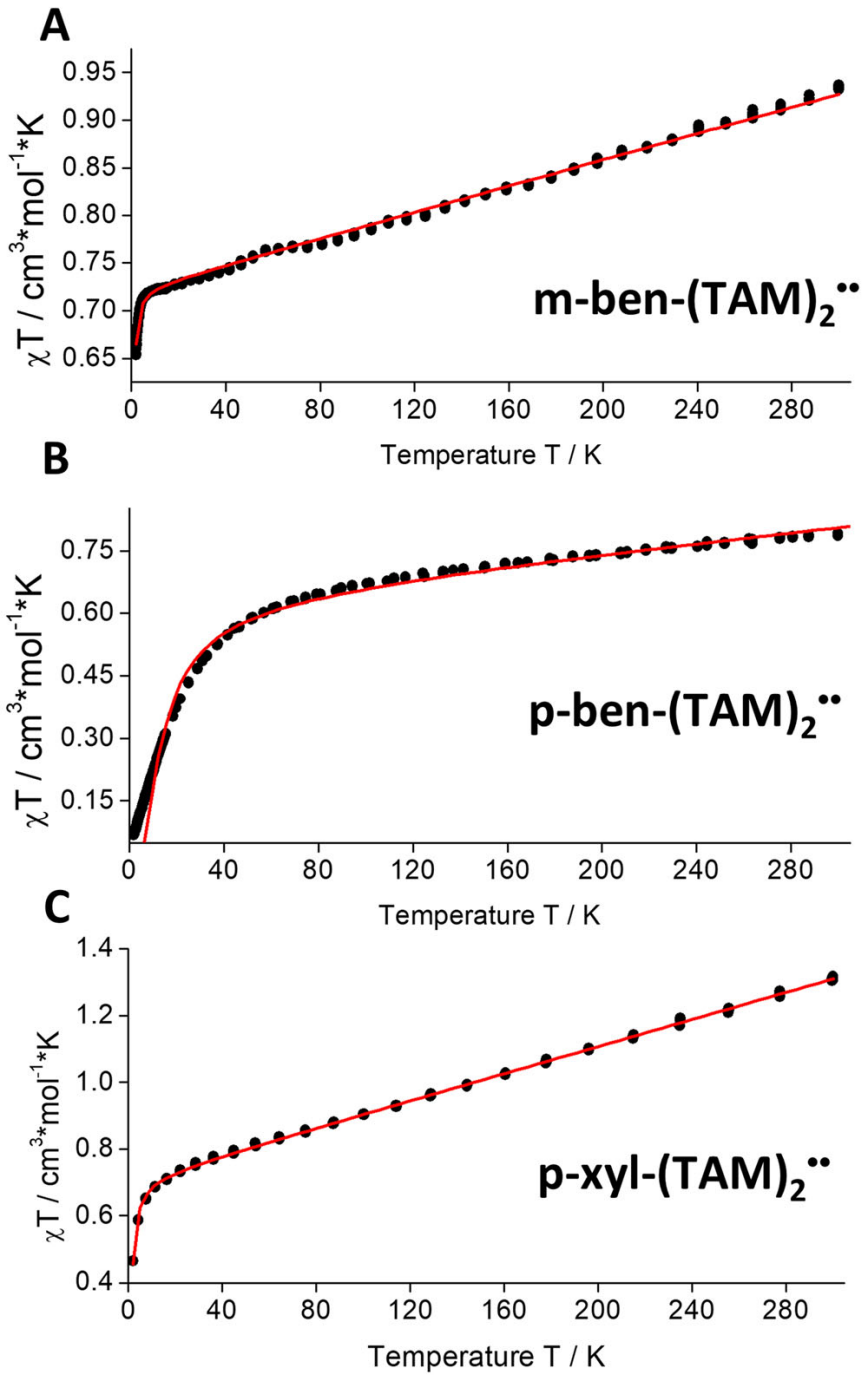
Plots of *χT* versus *T* from the VSM‐based magnetic susceptibility measurements at 10,000 Oe for (A) ***m*‐ben‐(TAM)_2_^••^**, (B) ***p*‐ben‐(TAM)_2_^••^**, and (C) ***p*‐xyl‐(TAM)_2_^••^**. The weak bump at around 60 K in (A) originates from residual elemental oxygen in the probe.

The prefactor *C* has an idealized value (CGS ‐units) of [43]

(6) $$C=\mu_{0}\;N_{A}\mu_{B}=-0.56\frac{cm^{3}}{mol},$$

where *N_A_* is Avogadro's number. For ***p*‐ben‐(TAM)_2_^••^**, the linear behavior of the plot changes at much higher temperatures, indicating that *J* is larger, but Equation (4) is also valid for this case [43]. The fits yielded *C* values that match the idealized value and *J*‐values of −4 GHz, −254 GHz, and −23 GHz for ***m*‐ben‐(TAM)_2_^••^**, ***p*‐ben‐(TAM)_2_^••^**, and ***p*‐xyl‐(TAM)_2_^••^,** respectively (Table 1). Comparing the *J*‐values found with EPR in frozen solution with those found by VSM in the powder, one realizes that: (1) *J* is antiferromagnetic in each case, and (2) *J* differs by two orders of magnitude for ***m*‐ben‐(TAM)_2_^••^**, while *J* is on the same order of magnitude for both methods for ***p*‐ben‐(TAM)_2_^••^** and ***p*‐xyl‐(TAM)_2_^••^**. Interestingly, performing EPR measurements on the same powder as used for the VSM measurement shows a very intense, broadened main field signal but no half‐field signal (Figure S8), indicating that *J* is indeed very small for ***m*‐ben‐(TAM)_2_^••^** in the powder. The broadening of the main field signal is not so large that it would explain the non‐detectability of a half‐field signal for a diradical with a *J* of −169 GHz [1, 45]. For example, a bistrityl with a *J* of −80 GHz still yielded a detectable half‐field signal [33]. To rationalize this large change in *J*, we performed DFT calculations and MD simulations.

### 2.4 DFT and MD Calculations

First, we optimized the structure of ***m*‐ben‐(TAM)_2_^••^** at the r^2^SCAN‐3c/def2‐mTZVPP level of theory as previously done for ***p*‐ben‐(TAM)_2_^••^** and ***p*‐xyl‐(TAM)_2_^••^** [46, 47]. The distance *r_C–C_* between the central carbon atoms of the trityls, the dihedral angles *θ* between the trityls and the linker group, and the opening angle *φ* between the trityl groups are anticipated to have the largest influence on *J* (Figure 5), and their values are collected in Table 2. For comparison with the *r_EPR_* obtained from the cw EPR measurements, we also calculated the inter‐spin distance *r*_spin_ *_–_*_spin_ by including the Mulliken spin‐density populations based on Equation (7) [31]

(7) $$\omega_{D}=\frac{\mu_{0}g^{2}\mu_{B}^{2}}{4\pi \hbar }\;\sum_{a=1}^{i}\sum_{b=i+1}^{N}\frac{1}{r_{ab}^{3}}\rho_{a}\rho_{b},$$

where *ρ_a_* and *ρ_b_* are the spin densities of an atom in the respective trityl site *a* or *b*, *r_ab_* is the inter‐atom distance, and $\omega_{D}$ is the electron–spin dipolar coupling.

**FIGURE 5:**
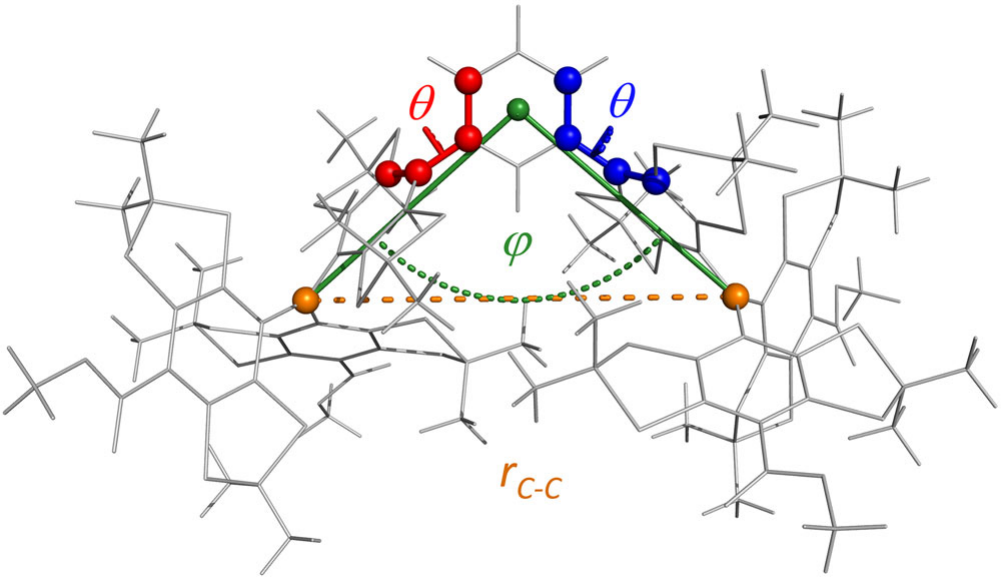
Visualization of the geometric parameters *r*, *φ*, and *θ*.

**TABLE 2:** Distances, angles, and *J*‐values in ***m*‐ben‐(TAM)_2_^••^**, ***p*‐ben‐(TAM)_2_^••^**, and ***p*‐xyl‐(TAM)_2_^••^** as obtained from the DFT calculations, MD(GFN2‐xTB) simulations at 300 K, and experimental measurements.

Comp.	*m*‐ben‐(TAM)_2_^••^		*p*‐ben‐(TAM)_2_^••^		*p*‐xyl‐(TAM)_2_^••^	
Method	DFT	MD	DFT	MD	DFT	MD
*θ*/°	75	61	63	57	80	90
*φ*/°	97	130	177	173	179	172
*r_C–C_*/Å	10.7	12.9	14.4	14.3	14.4	14.3
*r_spin–spin_*/Å	10.4	12.2	13.4	13.3	13.3	13.2
*r_EPR_*/Å	11.7		11.8		12.7	

*Note*: The experimental distances (*r*_EPR_), the calculated distances from DFT without (*r_C‐C,DFT_)* and with Mulliken spin density distributions taken into account (*r*_spin_ *_–_*_spin,DFT_), and the most probable distances from MD simulations without (*r*_C_ *_–_*_C,MD_) and with Mullikan spin density distributions taken into account (*r*_spin_ *_–_*_spin,MD_), the most probable dihedral angle *θ*, the most probable opening angle *φ*, and the experimental *J*‐values from VSM and EPR.

For ***p*‐xyl‐(TAM)_2_^••^**, the DFT‐derived distance *r*_spin_ *_–_*_spin,DFT_ matches the distance *r*_EPR_. For ***p*‐ben‐(TAM)_2_^••^**, *r*_EPR_ is significantly shorter than *r_C–C,DFT_* and *r*_spin_ *_–_*_spin,DFT_ due to the breakdown of the point‐dipole approximation as discussed previously [33]. However, for ***m*‐ben‐(TAM)_2_^••^**, *r_EPR_* is significantly longer than *r_C–C,DFT_* and *r_spin–spin,DFT_*, which is surprising. To test whether the structure of ***m*‐ben‐(TAM)_2_^••^** changes when dissolving the powder in toluene, we performed the DFT geometry optimization by including the CPCM solvation model for toluene, which changed the structural parameters only marginally (Chapter S6.1) [48, 49, 50, 51]. We then performed MD simulations for all three compounds on the GFN2‐xTB [52] level of theory, including the ALPB solvation model, and a bath temperature of 300 K (room temperature) and 150 K (close to the freezing point of the solvent), respectively [48, 49, 50, 51]. Fully converged conformational sampling at the r^2^SCAN‐3c level is prohibitively expensive for systems of this size. We therefore employed GFN2‐xTB for the MD simulations, which has been shown to yield reliable geometries and conformational energetics for organic π‐systems comparable to DFT [52].

Indeed, the opening angle *φ* increases for ***m*‐ben‐(TAM)_2_^••^** from 97° to 130° for in vacuo DFT to *in‐solution* MD at 300 K, respectively (Figure 6 and Table 2). This leads concomitantly to an increase of *r* to a most probable *r*_C_ *_–_*_C,MD_ of 12.9 Å, which, using the Mulliken spin‐density populations of the DFT structure, results in a *r*_spin_ *_–_*_spin,MD_ of 12.2 Å, which is in good agreement with the experimental distance of 11.7 Å (Figure 6 and Table 2). At the same time, its dihedral angle *θ* decreases from 75° to 61°. At 150 K, the distribution widths of the geometric parameters shrink, but the most probable distances remain as they were at 300 K (Chapter S6.2). In contrast, neither the distance *r_c–c_* nor the angles *φ* and *θ* change significantly for ***p*‐xyl‐(TAM)_2_^••^** and ***p*‐ben‐(TAM)_2_^••^** when going from the in vacuo DFT calculation to the *in‐solution* MD simulation (Figure 6 and Table 2). We assume in this discussion that the in vacuo DFT calculation resembles the structures in the powder (no solvent present), while shock‐freezing the EPR sample captures most conformers of the MD ensemble, as has been shown several times before [53, 54, 55]. This structural change in ***m*‐ben‐(TAM)_2_^••^** could also be the reason for the large difference between the *J*‐values in the powder and solution state. Fittingly, the structures and *J*‐values for ***p*‐ben‐(TAM)_2_^••^** and ***p*‐xyl‐(TAM)_2_^••^** change only marginally upon solvation. However, why the antiferromagnetic coupling increases when dissolving ***m*‐ben‐(TAM)_2_^••^** is harder to rationalize. Based on the A(^13^C)‐data, the spin density is the same for all three compounds at the connecting C‐atom of the trityl groups, that is, the para‐C. The large dihedral angle of *θ* = 75° found for the in vacuo DFT structure may diminish the ferromagnetic coupling through the π‐system to such an extent that it matches the antiferromagnetic coupling through the *σ*‐bonds, that is, both contributions almost cancel each other. This would explain the small antiferromagnetic coupling of −4 GHz observed in the powder. However, in the MD, that is, in solution, *θ* decreases to 61°, which should increase the contribution of the ferromagnetic π‐pathway and thus either decrease the overall antiferromagnetic coupling or make it overall ferromagnetic but not stronger antiferromagnetic. However, the simultaneous increase in *r* and *φ* may also diminish a through‐space contribution between the trityl groups, of which, the sign is hard to predict [56]. Interestingly, all three geometric changes bring the structure of ***m*‐ben‐(TAM)_2_^••^** closer to the structure of ***p*‐ben‐(TAM)_2_^••^**, yielding a similar magnitude of antiferromagnetic coupling. Thus, we speculate that the interplay of *r‐* and *φ*‐increase and *θ*‐decrease leads in ***m*‐ben‐(TAM)_2_^••^** to changes in the σ‐, π‐ and through‐space pathways such that *J* becomes considerably stronger antiferromagnetic in solution as compared to the powder. In addition, and because it was not possible to grow single crystals of any of the trityl radical compounds, we cannot rule out that *J* in the powder‐VSM measurements also contains intermolecular spin–spin coupling contributions. In solution, at micromolar concentrations, such intermolecular contributions are absent. One could try to shed light on this by calculating the *J*‐value of ***m*‐ben‐(TAM)_2_^••^** in the different structures and with and without the bridge by DFT [57]. However, it is difficult to reduce this broad conformational ensemble to a single representative structure (Chapter S6.4), and we cannot run DFT calculations for all 10^6^ conformers in the MD trajectory. Additionally, we and others have shown before that *J*‐values on the order of magnitude reported here, and the resulting differences in *J*‐values between the discussed diradicals, require an accuracy that is not given by unrestricted Kohn–Sham DFT methods [33, 58]. Furthermore, neglected averaging over the torsional degrees of freedom and effects of explicit solvent molecules may contribute to the uncertainty. We, therefore, calculated the *J*‐values only for the sake of completeness and show them only in the Supporting Information (Chapter S6.1).

**FIGURE 6:**
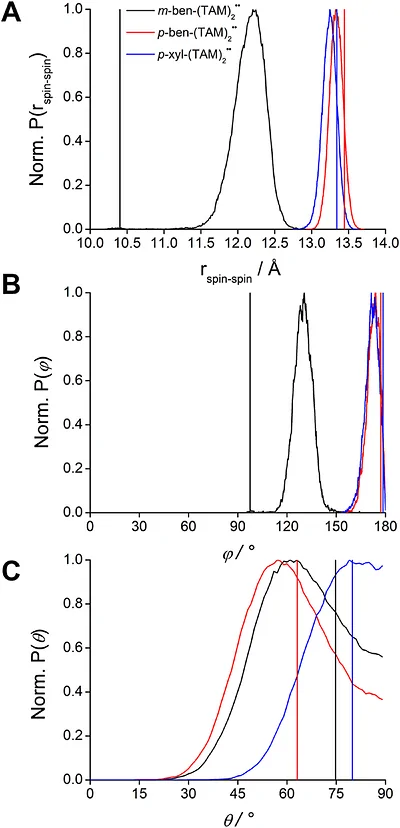
Plots of the MD‐derived structural parameters for ***m*‐ben‐(TAM)_2_^••^** (black), ***p*‐ben‐(TAM)_2_^••^** (red), and ***p*‐xyl‐(TAM)_2_^••^** (blue). (A) The distance *r*_spin_ *_–_*_spin_ obtained from the MD simulation, taking the spin density distribution into account. (B) The opening angle *φ*. (C) The dihedral angle *θ*. The vertical lines indicate, in each case, the values from the DFT structure.

## 3 Conclusion

We synthesized the *m*‐phenylene‐bridged diradical ***m*‐ben‐(TAM)_2_^••^** and obtained from VSM measurements a weak antiferromagnetic exchange coupling due to the large dihedral angle *θ* between the bridge plane and the trityl groups. In frozen solution, EPR revealed a significant increase in antiferromagnetic coupling, which goes, according to DFT and MD calculations, in parallel with large changes in the trityl–trityl distance *r*, the opening angle *φ* between the trityl groups, and the dihedral angle *θ*, when dissolving ***m*‐ben‐(TAM)_2_^••^**. For ***p*‐xyl‐(TAM)_2_^••^** and ***p*‐ben‐(TAM)_2_^••^**, the *in‐solution* and *in‐powder* measurements yielded *J*‐values on the same order of magnitude, and both do not undergo this structural change upon solvation. This study highlights that the tuning of *J* has to include more than just the topological rule and is an intricate interplay of topology, distances, and angles, and these are influenced by whether the molecule is in the powder state or in solution. This is particularly important if such diradicals are used in solution DNP or QIT solely based on solid state magnetometry or DFT calculations. These findings may serve as a basis for the rational design of new trityl‐based molecular magnets, materials for QIS, and DNP.

## Conflicts of Interest

The authors declare no conflicts of interest.

## Supporting information



The authors have cited additional references within the Supporting Information [59, 60, 61, 62, 63, 64, 65, 66, 67, 68, 69, 70, 71, 72, 73, 74, 75, 76, 77, 78, 79, 80, 81, 82, 83, 84].
**Supporting File 1**: chem71384‐sup‐0001‐SuppMat.docx

## Data Availability

The data that support the findings of this study are available from the corresponding author upon reasonable request.
