# Supplementary material for: Interplay of Bridge Topology and Conformation in Phenylene‐Bridged Trityl Diradicals
Source: Chemistry. 2026 Jul 9;32(39):e71384. doi: 10.1002/chem.71384 (PMC13715452; doi:10.1002/chem.71384)
Supplement: Supplementary file 1 [file CHEM-32-e71384-s001.docx]

**Interplay of Topology and Angles in Phenylene-bridged Trityl Diradicals**

Kevin Lars Kopp,^[a]^ Tobias Hett,^[a]^ Andrea Pellegrini,^[b]^ Stefan Grimme,^[c]^ and Olav Schiemann^[a]^*

^[a]^ Clausius-Institute for Physical and Theoretical Chemistry

University of Bonn

Wegelerstraße 12, 53115 Bonn, Germany

^[b]^ Department of Industrial Chemistry “Toso Montanari”

University of Bologna

Via P. Gobetti 85, 40129 Bologna, Italy

^[c]^ Mulliken Center, Clausius-Institute for Physical and Theoretical Chemistry

University of Bonn

Beringstraße 4, 53115 Bonn, Germany

E-mail: [Schiemann@uni-bonn.de](mailto:Schiemann@uni-bonn.de)

*Corresponding author

**Table of contents**

**1. General Procedures and Instrumentation**	3

**2. Synthesis**	3

2.1.	1,3-Bis[{Bis(8-methoxycarbonyl-2,2,6,6-tetramethylbenzo-[1,2-d;4,5-d′]-bis-[1,3]dithiol-4-yl)-(8-yl-2,2,6,6-tetramethylbenzo-[1,2-d;4,5-d′]-bis-[1,3]dithiol-4-yl)}-methanol]-benzene (**14**)	3

2.2.	1,3-Bis[{Bis(8-methoxycarbonyl-2,2,6,6-tetramethylbenzo-[1,2-d;4,5-d′]-bis-[1,3]dithiol-4-yl)-(8-yl-2,2,6,6-tetramethylbenzo-[1,2-d;4,5-d′]-bis-[1,3]dithiol-4-yl)}-methyl radical]-benzene (***m*-ben-(TAM)_2_^••^**)	3

**3. Analytical data**	4

3.1. NMR spectroscopy	4

3.2. Mass spectrometry	5

3.3. Medium pressure liquid chromatography on ***m*-ben-(TAM)_2_^••^**	6

**4. EPR spectroscopy**	7

**5. Temperature-dependent half-field measurement**	8

**6. Computational details**	9

6.1. General DFT procedures	9

6.2. Molecular dynamics simulation	10

6.3. Coordinates	21

6.4. DFT calculation of *J* on representative conformers from MD	32**7. References**	38

# **1. General Procedures and Instrumentation**

**NMR spectroscopy:** NMR spectra were recorded on an Avance III 500 spectrometer (Bruker BioSpin, Ettlingen, Germany). All chemical shifts are referenced to the residual hydrogen peaks of the deuterated NMR solvent.

**Mass spectrometry:** MALDI(+)-spectra were recorded on a Bruker Daltonics UltraFlex TOF/TOF spectrometer with *trans*-2-[-3(4-*tert*-Butylphenyl)-2-methyl-2-propenylidene]malononitrile (DCTB) as the matrix (Bruker Daltonics, Bremen, Germany). ESI-spectra were recorded on an Orbitrap XL mass spectrometer (Thermo Fisher Scientific, Bremen, Germany) with a Dionex Ultimate 3000 autosampler.

**EPR spectroscopy:** Continuous wave (cw) EPR measurements at X-band frequencies (~9.4 GHz) were performed on a Bruker EMXmicro EPR spectrometer (Bruker BioSpin, Ettlingen, Germany). Measurements at room temperature were done using a Bruker ER4119HS resonator, while for measurements down to 100 K, a Bruker ER4119HS resonator in combination with an ER4141VT temperature control system was used, which operates with a continuous flow of cold nitrogen gas. Measurements below 100 K were done using an ER4122SHQE resonator, an ER4122HV liquid helium cryostat, and a Mercury iTC503 temperature controller (sensor type ESR900, Oxford Instruments).

For the EPR measurements, a 50 µm solution of each of the radicals was prepared in toluene-d8, transferred into a 3 mm o.d. EPR tube (Wilmad LabGlass), degassed repeatedly, and flame-sealed under vacuum while still frozen.

The room temperature spectrum was recorded with a modulation amplitude of 0.2 G, 100 kHz modulation frequency, and 0.5792 mW microwave power. The conversion time was set to 20.22 ms and the receiver time constant to 10.24 ms. The spectrum at 100 K was recorded with a modulation amplitude of 0.4 G, 100 kHz modulation frequency, and 0.002333 mW microwave power. The conversion time was set to 30.10 ms, and the receiver time constant to 20.48 ms. The spectra were field-corrected using Mn^2+^-doped MgO.^[1]^

**VSM**: VSM measurements were performed on a Quantum Design (San Diego, USA) PPMS-VSM with a 9 T magnet. Samples were prepared within a polypropylene powder holder inside a brass tubing. The plots show the heating as well as the cooling curve merged into one graph for each of the three samples.

**Medium pressure liquid chromatography (MPLC):** The analytical MPLC was performed using a Sepacore X10 system from Büchi (Essen, Germany) and using Büchi Flashpure Select 12 g cartridges (Silica 25 µm spherical). For each run, a constant flow rate of 20 mL/min was used with tetrahydrofuran/n-hexane as the eluent. The sample was injected into the system by dissolving it in a minimal amount of dichloromethane.

**Synthesis:** Chemicals were purchased from commercial suppliers and used without further purification. Where indicated, solvents were degassed by applying three freeze-pump-thaw cycles. Thin-layer chromatography was conducted using 250 µm F254 silica plates provided by Merck, and spots were visualized with UV-light at 254 nm. Spots of trityl alcohols can be stained selectively by irradiating the TLC-plate for 5 min with UV-light at 254 nm (5 W). For column chromatography, silica gel (60 Å pore size, 40-63 µm particle size) purchased from Merck was used. Solvents were generally removed under reduced pressure by a rotary evaporator, and products were further dried in an oil-pump vacuum at 10^‑3^ mbar. Unless indicated otherwise, standard Schlenk techniques were used in the setup of the chemical reactions.

.

# **2. Synthesis**

## **2.1. 1,3-Bis[{Bis(8-methoxycarbonyl-2,2,6,6-tetramethylbenzo-[1,2-d;4,5-d′]-bis-[1,3]dithiol-4-yl)-(8-yl-2,2,6,6-tetramethylbenzo-[1,2-d;4,5-d′]-bis-[1,3]dithiol-4-yl)}-methanol]-benzene (14)**

Trityl alcohol **13** (100.0 mg, 0.0887 mmol), 1,3-Bis(4,4,5,5-tetramethyl-1,3,2-dioxaborolan-2-yl)-benzene (14.6 mg, 0.0442 mmol), Pd_2_dba_3_ (8.1 mg, 8.8 µmol), PPh_3_ (9.3 mg, 35 µmol), and TBAF (5.0 mg, 35 µmol) were placed in a flame-dried and argon-flushed Schlenk flask. The flask was evacuated and flushed again with argon. The solids were dissolved in degassed toluene (6 mL) and a degassed 2m solution of potassium carbonate (4 mL) was added. The reaction mixture was then stirred and heated to 90 °C for 2h. Afterwards, the reaction was allowed to cool to room temperature, and the phases were separated. The aqueous phase was extracted with DCM (3 x 20 mL). The combined organic phases were washed with water (2 x 20 mL) and brine (40 mL), dried over magnesium sulfate, and the solvents were removed under reduced pressure. The crude product was subjected to column chromatography on silica eluting with n-hexane/ethyl acetate (3:1 (v/v)) to obtain 72.8 mg (0.0350 mmol, 79%) (R_f_=0.34) of a yellow solid.

^1^H-NMR (500 MHz, CDCl_3_) δ: 7.55-7.42 (m, 3H), 7.20-7.12 (m, 1H), 6.73 (br. s, 2H), 3.96 (s, 12H), 1.83 (s, 6H), 1.82 (s, 3H), 1.79 (s, 12H), 1.78 (s, 9H), 1.71 (s, 12H), 1.70 (s, 6H), 1.66 (s, 18H), 1.59 (s, 6H).

^13^C-NMR (125 MHz, CDCl_3_) δ: 166.8, 166.8, 142.1, 141.8, 141.5, 140.9, 140.5, 139.2, 139.1, 138.7, 138.3, 135.2, 134.9, 133.0, 130.2, 129.4, 128.7, 121.1, 84.4, 62.8, 62.2, 62.1, 61.6, 60.9, 60.6, 52.5, 52.5, 35.1, 34.9, 34.7, 34.1, 33.4, 33.1, 31.6, 31.3, 30.9, 30.5, 30.3, 30.1, 29.8, 29.5, 28.1, 27.7, 27.5, 27.1.

HRMS (ESI+, m/z, [M]^+^): calc. for C_88_H_90_O_10_S_24_, 2075.9823; found 2075.9829.

## **2.2. 1,3-Bis[{Bis(8-methoxycarbonyl-2,2,6,6-tetramethylbenzo-[1,2-d;4,5-d′]-bis-[1,3]dithiol-4-yl)-(8-yl-2,2,6,6-tetramethylbenzo-[1,2-d;4,5-d′]-bis-[1,3]dithiol-4-yl)}-methyl radical]-benzene (m-ben-(TAM)_2_^••^)**

1,3-Bis[{Bis(8-methoxycarbonyl-2,2,6,6-tetramethylbenzo-[1,2-d;4,5-d′]-bis-[1,3]dithiol-4-yl)-(8-yl-2,2,6,6-tetramethylbenzo-[1,2-d;4,5-d′]-bis-[1,3]dithiol-4-yl)}-methanol]-benzene **14** (47.1 mg, 0.0227 mmol) was placed in a flame-dried and argon-flushed Schlenk flask. The flask was evacuated and flushed again with argon. The solid was then dissolved in 12 mL of DCM and cooled to 0 °C using an ice bath, and triflic acid (13.6 mg, 0.0907 mmol) was added. The reaction mixture was allowed to reach room temperature and was stirred for 6 hours. It was then cooled to 0 °C again, and tin(II)chloride (8.6 mg, 0.045 mmol) dissolved in a minimal amount of THF (~1 mL) was added. The reaction was again allowed to reach room temperature and was stirred for an additional 90 min. Afterwards, the reaction mixture was washed with water (3 × 10 mL) and brine (10 mL). The organic solution was dried over magnesium sulfate, and the solvent was removed under reduced pressure. The crude product was subjected to column chromatography on silica eluting with cyclohexane/ethyl acetate (3:1 (v/v)) to obtain 28.8 mg (0.0141 mmol, 62%) (R_f_=0.32) of a green solid.

HRMS (MALDI+, DCTB-Matrix, m/z, [M]^+^): calc. for C_88_H_88_O_8_S_24_, 2041.9766; found 2041.9745.

# **3. Analytical data**

## **3.1. NMR spectroscopy**

A small impurity is present in **14**, indicated by an additional signal in the ^1^H-NMR spectrum at 6.69 ppm. This has been shown previously.^[2]^

**Figure S1.** ^1^H-NMR of **14**, 500 MHz, CDCl_3_ (4% impurity).

**Figure S2.** ^13^C{^1^H}-NMR of **14**, 125 MHz, CDCl_3_.

## **3.2. Mass spectrometry**

**Figure S3.** ESI(+)-HRMS of **14**.

**Figure S4.** MALDI(+)-HRMS of ***m*-ben-(TAM)_2_^••^**.

The results of the mass spectrometry (Fig. S5) show slight oxidation based on the peak with +16 au and an additional peak in the mass spectrum, about 46.02 au heavier than the desired product. Both peaks have been assigned previously.^[2]^

**[M]^+^**

**[M+16]^+^**

**[M+46.02]^+^**

**Figure S5.** MALDI(+) mass spectrum of ***m*-ben-(TAM)_2_^••^**.

## **3.3. Medium pressure liquid chromatography on *m*-ben-(TAM)_2_^••^**

The purity of ***m*-ben-(TAM)_2_^••^** was assessed by analytical medium pressure liquid chromatography. The green line in the chromatogram shows the gradient percentage of tetrahydrofuran to n-hexane. The chromatogram around zero time was not shown because of the zero-adjustment of the detector. The signals around 8 minutes arise from the solvent system itself and are also visible without injection of a sample, which has been shown previously (also see figure S7).^[2]^

**Figure S6.** MPLC-chromatogram of ***m*-ben-(TAM)_2_^••^** (t = 10 min 49 sec).

**Figure S7:** MPLC-chromatogram without injection of sample.

# **4. EPR spectroscopy**

**Figure S8.** cw X-Band (9.46 GHz) EPR spectrum of **m-ben-(TAM)_2_^••^** at 100 K in solution (red) and as a solid powder (black). The inset shows the half-field (Δm_S_ = 2) EPR spectrum (at g = 4) at 7 K.

# **5. Temperature-dependent half-field measurement**

For data analysis, the cw EPR spectrum was integrated. The integrated spectrum was background-corrected by a polynomial of 3^rd^ order and then integrated once again to obtain the double integral of the experimental first-derivative spectrum, which is proportional to the magnetic susceptibility of the sample. The EPR measurement was performed in duplicates and the exchange-coupling constant obtained from the fits as well as its standard deviation is shown below the respective figures.

**Figure S9.** Bleaney-Bowers fit of the temperature-dependent half-field signal of biradical ***m*-ben-(TAM)_2_^••^** (50 µm, 86.41 mW, 1 G modulation amplitude, 56.3 ms conversion time, 40.96 ms time constant). *J* = -6.77±0.45 cm^-1^. The double-integral intensity of the half-field signal multiplied by the temperature is shown on the ordinate axis. Inset: Bleaney-Bowers fit with the double-integral intensity of the half-field signal shown on the ordinate axis.

**Figure S10.** Bleaney-Bowers fit of the temperature-dependent half-field signal of biradical **m-ben-(TAM)_2_^••^** (50 µm, 86.41 mW, 1 G modulation amplitude, 56.3 ms conversion time, 40.96 ms time const.). *J* = -4.52±0.36 cm^-1^. The double-integral intensity of the half-field signal multiplied by the temperature is shown on the ordinate axis. Inset: Bleaney-Bowers fit with the double-integral intensity of the half-field signal shown on the ordinate axis.

# **6. Computational details**

## **6.1. General DFT procedures**

All unrestricted Kohn-Sham (UKS) Density Functional Theory (DFT) calculations were performed with the ORCA^[3,4]^ quantum chemistry software package (version 6.0.1)^[5]^ using the SHARK integration^[6]^ algorithm. For calculation speed-up, the RIJCOSX approximation with def2/J as auxiliary basis was used.^[7–9]^ The SCF convergence criterion was set to “VeryTightSCF” to reliably achieve convergence of the energies and of the exchange-coupling constant *J*.

For the geometry optimization, the structure optimized using unrestricted Kohn-Sham (UKS) DFT. The optimization was done using r^2^SCAN-3c/def2-mTZVPP level of theory^[11]^ and with a D4 London dispersion correction.^[12–14]^ The structures were visualized using the ChimeraX software (version 11.1).^[15]^ Using the optimized structures, the exchange coupling constant *J* was determined by performing a broken symmetry calculation (keyword “BrokenSym 1,1” in the scf block) with the following convention:

$$J=-\frac{E_{\mathrm{HS}}-E_{\mathrm{BS}}}{\left\langle S^{2} \right\rangle_{\mathrm{HS}}-\left\langle S^{2} \right\rangle_{\mathrm{BS}}}$$

where *E_HS_* is the energy of the high-spin state and *E_BS_* is the energy of the broken-symmetry state. *J* < 0 corresponds to antiferromagnetic exchange coupling and *J* > 0 corresponds to ferromagnetic exchange coupling. The exchange coupling constant was determined using eight different functionals, i.e., M06L,^[16]^ TPSS,^[17]^ r^2^SCAN-3c, TPSSh-D4,^[17,18]^ B3LYP-D4,^[19]^ PBE0-D4,^[20–22]^ ωB97X-D4rev,^[23–25]^ and ωB97X-V.^[23,26]^ In each case a def2-TZVPP basis set was used.

**Table S1.** Calculated *J*-values (in GHz) of ***m*-ben-(TAM)_2_^••^**, ***p*-ben-(TAM)_2_^••^**, and ***p*-xyl-(TAM)_2_^••^** using functionals (FUNC) on the FUNC/def2-TZVPP//r^2^SCAN-3c/def2-mTZVPP level of theory.

|  | **FUNC** | | | | | | | |
| --- | --- | --- | --- | --- | --- | --- | --- | --- |
| **Comp.** | **M06L** | **TPSS** | **r^2^SCAN-3c** | **TPSSh-D4** | **B3LYP-D4** | **PBE0-D4** | **ωB97X-D4rev** | **ωB97X-V** |
| ***m*-ben-(TAM)_2_^••^** | -43.47 | -20.99 | -32.98 | -44.07 | -22.18 | 5.70 | -20.99 | -20.99 |
| ***p*-ben-(TAM)_2_^••^** | -236.54 | -318.98 | -299.79 | -221.25 | -198.76 | -169.98 | -128.01 | -150.20 |
| ***p*-xyl-(TAM)_2_^••^** | -42.87 | -57.26 | -41.67 | -15.29 | -29.68 | -3.90 | -31.18 | -37.17 |

Calculations have been rerun considering toluene as implicit solvent using CPCM, during the optimization and during the calculation of the J values.

**Table S2.** Geometrical parameters of ***m*-ben-(TAM)_2_^••^**, ***p*-ben-(TAM)_2_^••^**, and ***p*-xyl-(TAM)_2_^••^** considering or the implicit solvation.

|  | **Vacuum** | | | **[CPCM(Toluene)]** | | |  |
| --- | --- | --- | --- | --- | --- | --- | --- |
| **Comp.** | *r_C-C_* / Å | *θ* / ° | *φ / °* | *r_C-C_* / Å | *θ* / ° | *φ / °* | **RMSD** / Å |
| ***m*-ben-(TAM)_2_^••^** | 10.7 | 75 | 97 | 10.8 | 73 | 98 | 0.81 |
| ***p*-ben-(TAM)_2_^••^** | 14.4 | 63 | 177 | 14.4 | 65 | 178 | 0.26 |
| ***p*-xyl-(TAM)_2_^••^** | 14.4 | 80 | 179 | 14.4 | 82 | 180 | 0.38 |

**Table S3.** Calculated *J*-values (in GHz) of ***m*-ben-(TAM)_2_^••^**, ***p*-ben-(TAM)_2_^••^**, and ***p*-xyl-(TAM)_2_^••^** using functionals (FUNC) on the FUNC/def2-TZVPP [CPCM(Toluene)]//r^2^SCAN-3c/def2-mTZVPP [CPCM(Toluene)] level of theory.

|  | **FUNC** | | | | | | | |
| --- | --- | --- | --- | --- | --- | --- | --- | --- |
| **Comp.** | **M06L** | **TPSS** | **r^2^SCAN-3c** | **TPSSh-D4** | **B3LYP-D4** | **PBE0-D4** | **ωB97X-D4rev** | **ωB97X-V** |
| ***m*-ben-(TAM)_2_^••^** | 6.60 | 3.60 | -6.30 | -66.25 | -12.59 | -64.16 | -12.89 | -0.30 |
| ***p*-ben-(TAM)_2_^••^** | -184.67 | -268.01 | -394.23 | -230.54 | -146.60 | -208.06 | -111.22 | -112.72 |
| ***p*-xyl-(TAM)_2_^••^** | -13.79 | -21.88 | -168.48 | -65.95 | -6.90 | -72.55 | -22.18 | -21.88 |

## **6.2. Molecular dynamics simulation**

To assess the dynamics of the bistrityl compounds in solution, molecular dynamics (MD) simulations were run using the MD module in ORCA 6.1.1 with the DFT structure (see SI, section 6.1 for ***m*-ben-(TAM)_2_^••^** and reference ^[2]^ for ***p*-ben-(TAM)_2_^••^** and ***p*-xyl-(TAM)_2_^••^**) as input. Energies were calculated on the GFN2-xTB^[10]^-D4^[13,14]^ level of theory using 32 parallel processes on an AMD EPYC 7452 32-core processor and 252 GB RAM provided by Samsung M393A2K43DB3-CWE dual in-line memory modules (16 x 16 GB). The time step was set to 0.5 fs, the initial velocities were set corresponding to a temperature of either 300 K or 150 K, and the Nosé-Hoover chains (NHC)^[27,28]^ thermostat was used at a temperature of either 300 K or 150 K with a time constant of 10.0 fs. In total, the MD trajectories encompassed 10^6^ states, corresponding to a simulation time of 500 ps for each compound. Solvation of the molecules in toluene was simulated using the ddCOSMO solvation model.^[29–31]^

The distance *r_C–C_* between the central carbon atoms of the trityl moieties, the two dihedral angles *θ* between the trityl moieties and the phenylene bridge, and the opening angle *φ* (see Figure 5 of the main text) were calculated along the MD trajectory using a home-written PyMOL script.

In addition to calculating the distance *r_C–C_* between the central carbon atoms, the delocalization of the electron-spin density was taken into account by an extended point-dipole approach using eq. 7 in combination with eq. 3, both in the main text.^[32,33]^

The Mulliken spin population obtained from the DFT calculation (see SI, section 6.1) was used to approximate the electron-spin density at each atom. The interspin distance *r_spin-spin_* was calculated for each frame of the MD trajectory (shown as red traces in Figures S12-S20), and the distance distributions were obtained by histogram binning. The histograms for *r_spin-spin_*, θ, and *φ* are shown in Figure 6 in the main text. For the angular distributions of θ, the inherent symmetry was taken into account, and only the quadrant [0°, 90°] is shown. The MD trajectories (*r_spin-spin_*, θ, and *φ* as a function of the simulation time) and the raw histograms without symmetry considerations are shown in Figures S12-S20.

|  |  |
| --- | --- |
|  |  |
|  |  |

**Figure S11.** Molecular structure of **m-ben-(TAM)_2_^••^** (top), p**-ben-(TAM)_2_^••^** (center), **p-xyl-(TAM)_2_^••^** (bottom) optimized in vacuo (on the left) and using CPCM solvation model (on the right). The spin-density surfaces (iso-value 0.0025) are shown in blue.

**Figure S12.** Results from the trajectory and histograms of the 1^st^ MD simulation for **m-ben-(TAM)_2_^••^**. (A) Center-to-center distance r_C-C_. (B) 1^st^ dihedral angle θ_1_. (C) 2^nd^ dihedral angle θ_2_. (D) Opening angle ϕ.

**Figure S13.** Results from the trajectory and histograms of the 2^nd^ MD simulation for **m-ben-(TAM)_2_^••^**. (A) Center-to-center distance r_C-C_. (B) 1^st^ dihedral angle θ_1_. (C) 2^nd^ dihedral angle θ_2_. (D) Opening angle ϕ.

**Figure S14.** Results from the trajectory and histograms of the 3^rd^ MD simulation for **m-ben-(TAM)_2_^••^**. (A) Center-to-center distance r_C-C_. (B) 1^st^ dihedral angle θ_1_. (C) 2^nd^ dihedral angle θ_2_. (D) Opening angle ϕ.

**Figure S15.** Results from the trajectory and histograms of the 4^th^ MD simulation for **m-ben-(TAM)_2_^••^**. (A) Center-to-center distance r_C-C_. (B) 1^st^ dihedral angle θ_1_. (C) 2^nd^ dihedral angle θ_2_. (D) Opening angle ϕ.

**Figure S16.** Results from the trajectory and histograms of the 1^st^ MD simulation for **p-ben-(TAM)_2_^••^**. (A) Center-to-center distance r_C-C_. (B) 1^st^ dihedral angle θ_1_. (C) 2^nd^ dihedral angle θ_2_. (D) Opening angle ϕ.

**Figure S17.** Results from the trajectory and histograms of the 2^nd^ MD simulation for **p-ben-(TAM)_2_^••^**. (A) Center-to-center distance r_C-C_. (B) 1^st^ dihedral angle θ_1_. (C) 2^nd^ dihedral angle θ_2_. (D) Opening angle ϕ.

**Figure S18.** Results from the trajectory and histograms of the 1^st^ MD simulation for **p-xyl-(TAM)_2_^••^**. (A) Center-to-center distance r_C-C_. (B) 1^st^ dihedral angle θ_1_. (C) 2^nd^ dihedral angle θ_2_. (D) Opening angle ϕ.

**Figure S19.** Results from the trajectory and histograms of the 2^nd^ MD simulation for ***p*-xyl-(TAM)_2_^••^**. (A) Center-to-center distance r_C-C_. (B) 1^st^ dihedral angle *θ_1_*. (C) 2^nd^ dihedral angle *θ_2_*. (D) Opening angle *ϕ*.

**Figure S20.** Results from the trajectory and histograms of the MD simulation for ***m*-ben-(TAM)_2_^••^ at 150 K**. (A) Center-to-center distance r_C-C_. (B) 1^st^ dihedral angle *θ_1_*. (C) 2^nd^ dihedral angle *θ_2_*. (D) Opening angle *ϕ*.

## **6.3. Coordinates**

***m*-ben-(TAM)_2_^••^** In vacuum

```
C   -3.0657   4.7718   3.2842
C   -2.7188   3.9166   2.2283
C   -1.7226   4.2683   1.2934
C   -1.0351   5.4895   1.4896
C   -1.3651   6.3493   2.5410
C   -2.3686   5.9842   3.4737
S    0.2561   6.0175   0.4019
C    0.0225   7.8059   0.7868
S   -0.4534   7.8732   2.5744
S   -4.3277   4.1873   4.3721
C   -4.9053   2.8267   3.2411
S   -3.4328   2.3041   2.2675
C   -5.4000   1.6561   4.0838
C   -5.9923   3.3541   2.3084
C   -1.0947   8.3868  -0.0751
C    1.3388   8.5474   0.5883
C   -1.4183   3.4142   0.1611
C    2.1653   2.2505   0.2550
C    0.9276   2.7383   0.6887
C   -0.0452   3.1537  -0.2486
C    0.3361   3.1907  -1.6093
C    1.5738   2.6930  -2.0282
C    2.4855   2.1754  -1.1055
S   -0.6466   3.9329  -2.8722
C    0.1100   2.9300  -4.2351
S    1.8998   2.7465  -3.7654
S    3.2255   1.5739   1.5002
C    2.3749   2.4660   2.8870
S    0.5851   2.5300   2.4089
C    2.5273   1.6658   4.1736
C    2.9251   3.8818   3.0218
C    0.0024   3.6979  -5.5455
C   -0.5538   1.5602  -4.3128
C   -3.3733   0.8510  -1.8144
C   -2.3887   1.4400  -1.0133
C   -2.4896   2.7849  -0.6061
C   -3.6307   3.5110  -0.9966
C   -4.6275   2.9291  -1.7881
C   -4.5036   1.5903  -2.2108
S   -3.8688   5.2109  -0.5593
C   -5.0004   5.5577  -1.9798
S   -5.9723   4.0012  -2.2265
S   -3.0974  -0.8400  -2.2605
C   -1.9991  -1.1747  -0.8039
S   -1.0464   0.3885  -0.5371
C   -2.8418  -1.4959   0.4254
C   -1.0285  -2.2975  -1.1418
C   -4.1923   5.8805  -3.2320
C   -5.9473   6.6908  -1.6049
C    1.2250  -9.0290  -1.9592
C    1.6082  -8.0024  -1.0894
C    2.7794  -7.2530  -1.3207
C    3.5531  -7.5657  -2.4579
C    3.1810  -8.5936  -3.3301
C    2.0059  -9.3349  -3.0900
S    5.0546  -6.7101  -2.8453
C    5.7468  -8.1071  -3.8390
S    4.2923  -8.8807  -4.6835
S   -0.2728  -9.8756  -1.5469
C   -0.9401  -8.4696  -0.5382
S    0.5287  -7.6980   0.2790
C   -1.6326  -7.4516  -1.4384
C   -1.8793  -9.0175   0.5292
C    6.7027  -7.5556  -4.8896
C    6.4275  -9.1197  -2.9242
C    3.1857  -6.1970  -0.4079
C    3.3737  -2.9368  -2.3011
C    3.0108  -4.2452  -1.9605
C    3.6704  -4.9241  -0.9109
C    4.7537  -4.2664  -0.2834
C    5.1226  -2.9717  -0.6561
C    4.4138  -2.2751  -1.6396
S    5.6905  -4.9765   1.0376
C    7.1550  -3.8716   0.7685
S    6.4467  -2.2342   0.2611
S    2.3776  -2.1293  -3.5251
C    1.7243  -3.7214  -4.2180
S    1.5555  -4.8551  -2.7611
C    0.3557  -3.4874  -4.8440
C    2.7148  -4.3096  -5.2169
C    8.0384  -4.4311  -0.3416
C    7.9142  -3.7157   2.0795
C    2.3686  -5.6201   3.2472
C    2.5000  -5.4129   1.8682
C    3.0771  -6.3973   1.0336
C    3.5357  -7.5884   1.6397
C    3.3959  -7.8125   3.0155
C    2.8138  -6.8224   3.8350
S    4.4719  -8.7929   0.7478
C    4.0661 -10.1570   1.9201
S    4.0328  -9.3613   3.5956
S    1.5558  -4.3372   4.1501
C    1.8059  -3.0491   2.8352
S    1.7316  -3.9575   1.2305
C    0.6666  -2.0385   2.9004
C    3.1675  -2.3809   2.9940
C    5.1744 -11.2026   1.8859
C    2.7044 -10.7559   1.5847
C    4.7968   1.9544  -2.1424
C    3.6632   1.3958  -1.5539
C    3.5817   0.0127  -1.4053
C    4.6107  -0.8203  -1.8401
C    5.7483  -0.2549  -2.4184
C    5.8335   1.1262  -2.5667
C   -2.6726   6.7573   4.6899
O   -3.5323   6.4575   5.4984
O   -1.8653   7.8249   4.8582
C   -2.1101   8.6040   6.0446
C   -5.4901   0.9297  -3.0860
O   -5.2281   0.0196  -3.8474
O   -6.7329   1.4341  -2.9367
C   -7.7279   0.8899  -3.8266
C    1.5652 -10.4463  -3.9532
O    0.8709 -11.3706  -3.5780
O    2.0007 -10.3193  -5.2241
C    1.6729 -11.4174  -6.0987
C    2.6141  -6.9922   5.2868
O    1.7996  -6.3730   5.9434
O    3.4497  -7.9039   5.8250
C    3.2529  -8.1678   7.2282
H   -6.2498   1.9741   4.6978
H   -4.6140   1.2751   4.7402
H   -5.7470   0.8460   3.4327
H   -5.6230   4.1892   1.7063
H   -6.8517   3.6973   2.8962
H   -6.3189   2.5604   1.6263
H   -0.8231   8.3138  -1.1346
H   -1.2580   9.4396   0.1827
H   -2.0273   7.8367   0.0798
H    2.1329   8.1230   1.2077
H    1.2146   9.6052   0.8452
H    1.6408   8.4937  -0.4634
H    3.5851   1.6061   4.4517
H    2.1337   0.6527   4.0607
H    1.9952   2.1670   4.9893
H    2.7893   4.4440   2.0936
H    3.9937   3.8449   3.2623
H    2.3943   4.4111   3.8214
H   -1.0523   3.8288  -5.8116
H    0.4780   3.1269  -6.3501
H    0.4784   4.6793  -5.4770
H   -0.4601   1.0206  -3.3661
H   -0.0809   0.9639  -5.1018
H   -1.6213   1.6715  -4.5377
H   -3.4138  -2.4151   0.2548
H   -3.5405  -0.6833   0.6458
H   -2.1904  -1.6373   1.2952
H   -0.3677  -2.0274  -1.9695
H   -1.5768  -3.2093  -1.4040
H   -0.4076  -2.5368  -0.2719
H   -3.5858   6.7781  -3.0631
H   -4.8668   6.0573  -4.0777
H   -3.5208   5.0548  -3.4836
H   -6.5181   6.4532  -0.7036
H   -6.6449   6.8834  -2.4275
H   -5.3767   7.6099  -1.4318
H   -2.4794  -7.9210  -1.9521
H   -0.9404  -7.0603  -2.1897
H   -1.9998  -6.6116  -0.8374
H   -1.3721  -9.7414   1.1721
H   -2.7385  -9.5049   0.0554
H   -2.2600  -8.1979   1.1483
H    6.2056  -6.8297  -5.5381
H    7.0940  -8.3734  -5.5047
H    7.5549  -7.0708  -4.4007
H    6.8134  -9.9579  -3.5158
H    5.7235  -9.5085  -2.1830
H    7.2591  -8.6433  -2.3923
H    0.4501  -2.8012  -5.6924
H   -0.3535  -3.0649  -4.1283
H   -0.0440  -4.4334  -5.2251
H    3.6886  -4.4805  -4.7498
H    2.8409  -3.6222  -6.0613
H    2.3429  -5.2719  -5.5876
H    8.4168  -5.4179  -0.0517
H    8.8860  -3.7593  -0.5189
H    7.4724  -4.5382  -1.2714
H    7.2718  -3.3134   2.8669
H    8.7673  -3.0434   1.9377
H    8.3044  -4.6873   2.4011
H    0.6766  -1.5286   3.8702
H   -0.3043  -2.5247   2.7750
H    0.7917  -1.2704   2.1275
H    3.9777  -3.1105   2.9020
H    3.2315  -1.9050   3.9801
H    3.3025  -1.6137   2.2223
H    5.2205 -11.6602   0.8916
H    4.9580 -11.9988   2.6065
H    6.1470 -10.7641   2.1231
H    1.9222  -9.9919   1.6082
H    2.4548 -11.5407   2.3081
H    2.7226 -11.1894   0.5779
H    4.8683   3.0319  -2.2622
H    2.6938  -0.4231  -0.9525
H    6.5570  -0.8968  -2.7550
H    6.7171   1.5633  -3.0224
H   -1.3797   9.4129   6.0124
H   -1.9709   7.9890   6.9380
H   -3.1293   8.9999   6.0370
H   -8.6482   1.4259  -3.5936
H   -7.8467  -0.1826  -3.6500
H   -7.4364   1.0524  -4.8680
H    2.1252 -11.1671  -7.0586
H    0.5879 -11.5143  -6.1932
H    2.0842 -12.3517  -5.7067
H    3.9817  -8.9380   7.4816
H    2.2341  -8.5215   7.4093
H    3.4271  -7.2598   7.8118
```

***m*-ben-(TAM)_2_^••^** In CPCM(Toluene)

```
C   -3.0584   4.8554   3.2724
C   -2.7317   3.9901   2.2183
C   -1.7386   4.3235   1.2737
C   -1.0406   5.5407   1.4524
C   -1.3494   6.4101   2.5032
C   -2.3449   6.0604   3.4502
S    0.2404   6.0526   0.3449
C    0.0341   7.8482   0.7225
S   -0.4192   7.9232   2.5170
S   -4.3165   4.2856   4.3753
C   -4.9259   2.9282   3.2567
S   -3.4665   2.3865   2.2717
C   -5.4289   1.7674   4.1070
C   -6.0129   3.4679   2.3317
C   -1.0866   8.4402  -0.1259
C    1.3568   8.5723   0.5053
C   -1.4443   3.4521   0.1502
C    2.1211   2.2327   0.2758
C    0.8890   2.7445   0.6980
C   -0.0730   3.1650  -0.2488
C    0.3162   3.1847  -1.6079
C    1.5536   2.6759  -2.0136
C    2.4526   2.1513  -1.0818
S   -0.6530   3.9257  -2.8826
C    0.1152   2.9240  -4.2426
S    1.8982   2.7300  -3.7480
S    3.1565   1.5357   1.5305
C    2.3194   2.4423   2.9169
S    0.5350   2.5599   2.4186
C    2.4346   1.6293   4.1990
C    2.9080   3.8404   3.0661
C    0.0273   3.6959  -5.5516
C   -0.5547   1.5582  -4.3321
C   -3.4583   0.8907  -1.7706
C   -2.4508   1.4773  -0.9966
C   -2.5255   2.8302  -0.6093
C   -3.6568   3.5703  -1.0037
C   -4.6749   2.9906  -1.7698
C   -4.5828   1.6405  -2.1637
S   -3.8579   5.2834  -0.6022
C   -5.0010   5.6256  -2.0168
S   -6.0041   4.0798  -2.2135
S   -3.2165  -0.8141  -2.1867
C   -2.0989  -1.1443  -0.7427
S   -1.1192   0.4108  -0.5216
C   -2.9253  -1.4340   0.5048
C   -1.1507  -2.2867  -1.0786
C   -4.2050   5.9052  -3.2862
C   -5.9206   6.7841  -1.6533
C    1.2859  -9.0869  -1.9976
C    1.6521  -8.0612  -1.1194
C    2.8195  -7.3007  -1.3366
C    3.6067  -7.6029  -2.4676
C    3.2492  -8.6282  -3.3496
C    2.0781  -9.3802  -3.1238
S    5.1058  -6.7359  -2.8376
C    5.8200  -8.1187  -3.8395
S    4.3728  -8.8957  -4.6968
S   -0.2100  -9.9459  -1.6003
C   -0.9041  -8.5498  -0.5942
S    0.5553  -7.7732   0.2391
C   -1.5942  -7.5353  -1.4987
C   -1.8488  -9.1089   0.4617
C    6.7756  -7.5494  -4.8802
C    6.5040  -9.1335  -2.9309
C    3.2083  -6.2442  -0.4166
C    3.3694  -2.9650  -2.2781
C    3.0229  -4.2825  -1.9557
C    3.6851  -4.9635  -0.9089
C    4.7581  -4.2996  -0.2702
C    5.1139  -2.9968  -0.6284
C    4.4003  -2.2984  -1.6072
S    5.7018  -5.0119   1.0452
C    7.1575  -3.8871   0.7975
S    6.4260  -2.2541   0.3015
S    2.3601  -2.1530  -3.4887
C    1.7271  -3.7409  -4.2115
S    1.5804  -4.9028  -2.7716
C    0.3537  -3.5169  -4.8290
C    2.7237  -4.2951  -5.2227
C    8.0541  -4.4216  -0.3131
C    7.9056  -3.7347   2.1147
C    2.3495  -5.6882   3.2327
C    2.4882  -5.4765   1.8554
C    3.0876  -6.4501   1.0238
C    3.5596  -7.6347   1.6316
C    3.4196  -7.8594   3.0078
C    2.8145  -6.8808   3.8242
S    4.5156  -8.8261   0.7432
C    4.1412 -10.1971   1.9210
S    4.0893  -9.3927   3.5928
S    1.5032  -4.4191   4.1277
C    1.7467  -3.1222   2.8196
S    1.6983  -4.0334   1.2134
C    0.5962  -2.1251   2.8767
C    3.0991  -2.4402   2.9922
C    5.2705 -11.2191   1.8902
C    2.7920 -10.8242   1.5882
C    4.7637   1.9330  -2.1133
C    3.6331   1.3705  -1.5208
C    3.5573  -0.0127  -1.3657
C    4.5893  -0.8417  -1.8025
C    5.7232  -0.2738  -2.3870
C    5.8033   1.1079  -2.5382
C   -2.6213   6.8450   4.6672
O   -3.4843   6.5643   5.4824
O   -1.7909   7.8898   4.8252
C   -2.0014   8.6889   6.0105
C   -5.6020   0.9807  -3.0032
O   -5.3682   0.0541  -3.7575
O   -6.8287   1.5028  -2.8297
C   -7.8660   0.9638  -3.6816
C    1.6566 -10.4896  -4.0014
O    0.9834 -11.4341  -3.6305
O    2.0805 -10.3356  -5.2677
C    1.7763 -11.4262  -6.1678
C    2.6130  -7.0584   5.2762
O    1.7637  -6.4726   5.9235
O    3.4822  -7.9274   5.8187
C    3.2960  -8.2081   7.2251
H   -6.2705   2.1003   4.7239
H   -4.6440   1.3770   4.7594
H   -5.7900   0.9617   3.4584
H   -5.6385   4.2965   1.7232
H   -6.8623   3.8222   2.9270
H   -6.3561   2.6730   1.6592
H   -0.8272   8.3615  -1.1879
H   -1.2273   9.4968   0.1293
H   -2.0272   7.9077   0.0429
H    2.1553   8.1412   1.1146
H    1.2454   9.6316   0.7606
H    1.6413   8.5115  -0.5507
H    3.4882   1.5378   4.4840
H    2.0109   0.6292   4.0777
H    1.9110   2.1445   5.0113
H    2.8075   4.4103   2.1379
H    3.9701   3.7684   3.3258
H    2.3814   4.3788   3.8622
H   -1.0241   3.8341  -5.8260
H    0.5074   3.1209  -6.3506
H    0.5094   4.6739  -5.4770
H   -0.4838   1.0197  -3.3824
H   -0.0699   0.9608  -5.1129
H   -1.6151   1.6805  -4.5822
H   -3.5108  -2.3484   0.3560
H   -3.6100  -0.6094   0.7256
H   -2.2602  -1.5744   1.3642
H   -0.5214  -2.0508  -1.9407
H   -1.7200  -3.1992  -1.2874
H   -0.5015  -2.4955  -0.2216
H   -3.5832   6.7966  -3.1433
H   -4.8902   6.0796  -4.1236
H   -3.5524   5.0628  -3.5336
H   -6.4846   6.5783  -0.7399
H   -6.6233   6.9716  -2.4725
H   -5.3270   7.6933  -1.5084
H   -2.4351  -8.0108  -2.0163
H   -0.9006  -7.1376  -2.2457
H   -1.9730  -6.7010  -0.8972
H   -1.3437  -9.8303   1.1093
H   -2.6981  -9.6004  -0.0251
H   -2.2418  -8.2919   1.0761
H    6.2776  -6.8213  -5.5257
H    7.1756  -8.3605  -5.4983
H    7.6202  -7.0637  -4.3796
H    6.9020  -9.9596  -3.5312
H    5.8021  -9.5390  -2.1963
H    7.3302  -8.6530  -2.3945
H    0.4368  -2.8100  -5.6614
H   -0.3610  -3.1245  -4.1017
H   -0.0277  -4.4621  -5.2301
H    3.7044  -4.4520  -4.7647
H    2.8295  -3.5933  -6.0578
H    2.3644  -5.2568  -5.6066
H    8.4439  -5.4057  -0.0292
H    8.8951  -3.7373  -0.4717
H    7.4996  -4.5237  -1.2506
H    7.2544  -3.3504   2.9040
H    8.7494  -3.0492   1.9819
H    8.3078  -4.7051   2.4246
H    0.6029  -1.6089   3.8429
H   -0.3702  -2.6195   2.7481
H    0.7200  -1.3645   2.0963
H    3.9183  -3.1619   2.9184
H    3.1424  -1.9527   3.9734
H    3.2335  -1.6783   2.2152
H    5.3246 -11.6763   0.8963
H    5.0670 -12.0168   2.6127
H    6.2346 -10.7618   2.1266
H    1.9934 -10.0768   1.6096
H    2.5615 -11.6122   2.3141
H    2.8241 -11.2623   0.5838
H    4.8310   3.0105  -2.2374
H    2.6728  -0.4515  -0.9087
H    6.5321  -0.9130  -2.7290
H    6.6837   1.5475  -2.9978
H   -1.2518   9.4784   5.9590
H   -1.8610   8.0779   6.9059
H   -3.0099   9.1101   6.0082
H   -8.7651   1.5257  -3.4299
H   -8.0035  -0.1009  -3.4764
H   -7.6013   1.1041  -4.7329
H    2.2247 -11.1455  -7.1206
H    0.6938 -11.5396  -6.2668
H    2.2105 -12.3566  -5.7929
H    4.0566  -8.9482   7.4727
H    2.2935  -8.6070   7.4003
H    3.4364  -7.2962   7.8111
```

***p*-ben-(TAM)_2_^••^** in vacuum

```
C    2.1617   9.7262   1.6739
C    1.0465   9.0179   1.2131
C    1.1899   7.9305   0.3283
C    2.4933   7.5757  -0.0771
C    3.6142   8.2806   0.3743
C    3.4584   9.3630   1.2633
S    2.8032   6.2536  -1.2144
C    4.4159   6.9549  -1.7846
S    5.1691   7.7331  -0.2826
S    1.8287  11.0586   2.7908
C    0.2011  10.3575   3.3345
S   -0.5222   9.5587   1.8316
C    0.4128   9.3183   4.4307
C   -0.7100  11.4895   3.7920
C    5.3148   5.8263  -2.2732
C    4.1825   8.0071  -2.8633
C    0.0325   7.1926  -0.1571
C    0.6000   3.5721   0.7911
C    0.5871   4.9696   0.8530
C    0.0641   5.7410  -0.2122
C   -0.4502   5.0393  -1.3278
C   -0.4732   3.6410  -1.3559
C    0.0574   2.8843  -0.3033
S   -0.9071   5.8430  -2.8299
C   -1.9399   4.4264  -3.4278
S   -1.0548   2.8989  -2.8540
S    1.1632   2.7273   2.2423
C    2.0537   4.2058   2.9213
S    1.0365   5.6660   2.4121
C    2.0951   4.1239   4.4417
C    3.4491   4.3076   2.3143
C   -1.9921   4.4439  -4.9498
C   -3.3302   4.4981  -2.8053
C   -3.6079   8.1673  -0.4612
C   -2.4491   7.4655  -0.1158
C   -1.1777   7.9088  -0.5393
C   -1.1170   9.0621  -1.3477
C   -2.2675   9.7918  -1.6818
C   -3.5355   9.3284  -1.2660
S    0.3747   9.5330  -2.1689
C   -0.1558  11.2777  -2.4179
S   -1.9867  11.2048  -2.7128
S   -5.1239   7.5119   0.1765
C   -4.3008   6.5716   1.5485
S   -2.6670   6.0262   0.8861
C   -4.0993   7.4793   2.7586
C   -5.1423   5.3484   1.8927
C    0.5280  11.8498  -3.6544
C    0.1406  12.1019  -1.1687
C   -2.1713  -9.7200   1.6700
C   -1.0529  -9.0138   1.2140
C   -1.1905  -7.9266   0.3279
C   -2.4914  -7.5697  -0.0834
C   -3.6155  -8.2725   0.3634
C   -3.4655  -9.3549   1.2535
S   -2.7939  -6.2473  -1.2223
C   -4.4056  -6.9456  -1.7991
S   -5.1665  -7.7224  -0.3005
S   -1.8456 -11.0525   2.7891
C   -0.2191 -10.3542   3.3396
S    0.5122  -9.5570   1.8397
C   -0.4336  -9.3145   4.4346
C    0.6880 -11.4878   3.8011
C   -5.3003  -5.8151  -2.2914
C   -4.1698  -7.9981  -2.8771
C   -0.0295  -7.1910  -0.1526
C   -0.5968  -3.5694   0.7918
C   -0.5851  -4.9669   0.8545
C   -0.0585  -5.7393  -0.2082
C    0.4614  -5.0387  -1.3217
C    0.4851  -3.6405  -1.3508
C   -0.0496  -2.8828  -0.3010
S    0.9249  -5.8438  -2.8210
C    1.9603  -4.4279  -3.4160
S    1.0732  -2.8997  -2.8470
S   -1.1653  -2.7235   2.2402
C   -2.0592  -4.2010   2.9167
S   -1.0412  -5.6622   2.4122
C   -2.1063  -4.1181   4.4369
C   -3.4525  -4.3022   2.3046
C    2.0184  -4.4467  -4.9378
C    3.3481  -4.4991  -2.7880
C    3.6101  -8.1741  -0.4368
C    2.4513  -7.4686  -0.0991
C    1.1810  -7.9095  -0.5288
C    1.1220  -9.0636  -1.3362
C    2.2724  -9.7967  -1.6629
C    3.5393  -9.3362  -1.2402
S   -0.3662  -9.5316  -2.1652
C    0.1610 -11.2780  -2.4091
S    1.9938 -11.2104  -2.6935
S    5.1241  -7.5218   0.2089
C    4.2956  -6.5769   1.5744
S    2.6672  -6.0281   0.9015
C    4.0843  -7.4821   2.7848
C    5.1385  -5.3555   1.9216
C   -0.5171 -11.8498  -3.6488
C   -0.1447 -12.0998  -1.1606
C    0.0320   1.4029  -0.3319
C   -1.1868   0.7183  -0.3431
C   -1.2144  -0.6686  -0.3348
C   -0.0240  -1.4013  -0.3308
C    1.1949  -0.7168  -0.3378
C    1.2225   0.6701  -0.3306
C    4.5919  10.1642   1.7610
O    4.5067  11.3223   2.1201
O    5.7446   9.4633   1.7968
C    6.9094  10.2162   2.1899
C   -4.8099   9.9282  -1.7001
O   -5.8987   9.4093  -1.5342
O   -4.6597  11.1083  -2.3343
C   -5.8747  11.7000  -2.8335
C   -4.6026 -10.1541   1.7463
O   -4.5208 -11.3120   2.1064
O   -5.7544  -9.4513   1.7765
C   -6.9221 -10.2022   2.1646
C    4.8146  -9.9403  -1.6657
O    5.9039  -9.4244  -1.4938
O    4.6649 -11.1207  -2.2995
C    5.8812 -11.7165  -2.7905
H    0.8688   9.7894   5.3090
H    1.0697   8.5163   4.0822
H   -0.5485   8.8770   4.7189
H   -0.8619  12.2264   2.9991
H   -0.2726  11.9904   4.6626
H   -1.6832  11.0869   4.0941
H    5.4755   5.0772  -1.4936
H    6.2847   6.2295  -2.5843
H    4.8616   5.3405  -3.1442
H    5.1397   8.4386  -3.1780
H    3.5418   8.8103  -2.4882
H    3.6918   7.5506  -3.7304
H    2.6692   3.2439   4.7513
H    1.0906   4.0597   4.8671
H    2.5993   5.0084   4.8461
H    3.3983   4.3787   1.2240
H    4.0392   3.4248   2.5864
H    3.9528   5.2065   2.6886
H   -2.4907   5.3573  -5.2916
H   -2.5767   3.5908  -5.3108
H   -0.9914   4.3989  -5.3864
H   -3.2728   4.4971  -1.7130
H   -3.9286   3.6395  -3.1312
H   -3.8301   5.4227  -3.1166
H   -5.0694   7.8365   3.1233
H   -3.4823   8.3438   2.4973
H   -3.5964   6.9261   3.5604
H   -5.2797   4.7018   1.0223
H   -6.1260   5.6638   2.2576
H   -4.6591   4.7738   2.6906
H    1.6114  11.8844  -3.4955
H    0.1865  12.8763  -3.8267
H    0.3161  11.2511  -4.5435
H   -0.3512  11.6743  -0.2903
H   -0.2135  13.1300  -1.3070
H    1.2200  12.1175  -0.9780
H   -0.8945  -9.7844   5.3110
H   -1.0874  -8.5112   4.0832
H    0.5272  -8.8749   4.7270
H    0.8419 -12.2250   3.0089
H    0.2460 -11.9879   4.6699
H    1.6606 -11.0869   4.1073
H   -5.4627  -5.0659  -1.5122
H   -6.2698  -6.2165  -2.6063
H   -4.8427  -5.3301  -3.1604
H   -5.1265  -8.4276  -3.1958
H   -3.5322  -8.8026  -2.4994
H   -3.6744  -7.5426  -3.7420
H   -2.6808  -3.2375   4.7438
H   -1.1034  -4.0545   4.8660
H   -2.6127  -5.0020   4.8399
H   -3.3978  -4.3740   1.2145
H   -4.0430  -3.4188   2.5739
H   -3.9582  -5.2004   2.6776
H    2.5182  -5.3605  -5.2768
H    2.6046  -3.5941  -5.2972
H    1.0194  -4.4019  -5.3782
H    3.2865  -4.4970  -1.6959
H    3.9478  -3.6408  -3.1124
H    3.8492  -5.4239  -3.0965
H    5.0512  -7.8414   3.1559
H    3.4665  -8.3453   2.5211
H    3.5782  -6.9262   3.5826
H    5.2832  -4.7109   1.0509
H    6.1190  -5.6732   2.2933
H    4.6520  -4.7781   2.7154
H   -1.6015 -11.8815  -3.4961
H   -0.1773 -12.8774  -3.8181
H   -0.2986 -11.2526  -4.5374
H    0.3431 -11.6725  -0.2800
H    0.2074 -13.1291  -1.2956
H   -1.2253 -12.1124  -0.9762
H   -2.1144   1.2830  -0.3501
H   -2.1642  -1.1950  -0.3280
H    2.1225  -1.2814  -0.3406
H    2.1722   1.1966  -0.3204
H    7.7416   9.5148   2.1268
H    6.7914  10.5896   3.2108
H    7.0614  11.0618   1.5134
H   -5.5626  12.6179  -3.3325
H   -6.5574  11.9196  -2.0081
H   -6.3674  11.0223  -3.5362
H   -7.7530  -9.4997   2.0971
H   -6.8095 -10.5750   3.1864
H   -7.0721 -11.0482   1.4881
H    5.5696 -12.6342  -3.2901
H    6.5583 -11.9367  -1.9607
H    6.3800 -11.0411  -3.4911
```

***p*-ben-(TAM)_2_^••^** in CPCM(Toluene)

```
C    2.1335   9.7420   1.7161
C    1.0311   9.0244   1.2393
C    1.1969   7.9369   0.3579
C    2.5091   7.5911  -0.0260
C    3.6174   8.3050   0.4434
C    3.4394   9.3883   1.3273
S    2.8501   6.2703  -1.1557
C    4.4703   6.9792  -1.7020
S    5.1870   7.7658  -0.1849
S    1.7676  11.0720   2.8269
C    0.1372  10.3584   3.3508
S   -0.5512   9.5522   1.8334
C    0.3429   9.3235   4.4510
C   -0.7912  11.4823   3.7916
C    5.3850   5.8553  -2.1702
C    4.2509   8.0278  -2.7862
C    0.0514   7.1928  -0.1471
C    0.5887   3.5749   0.8308
C    0.5850   4.9732   0.8830
C    0.0872   5.7406  -0.1975
C   -0.4011   5.0345  -1.3222
C   -0.4257   3.6362  -1.3442
C    0.0715   2.8836  -0.2730
S   -0.8207   5.8293  -2.8410
C   -1.8350   4.4083  -3.4659
S   -0.9626   2.8864  -2.8552
S    1.1019   2.7353   2.3033
C    1.9920   4.2067   3.0009
S    1.0081   5.6765   2.4470
C    1.9880   4.1368   4.5219
C    3.4050   4.2857   2.4337
C   -1.8435   4.4181  -4.9882
C   -3.2417   4.4808  -2.8829
C   -3.5863   8.1459  -0.5499
C   -2.4321   7.4596  -0.1605
C   -1.1529   7.9036  -0.5595
C   -1.0784   9.0482  -1.3788
C   -2.2251   9.7648  -1.7532
C   -3.5012   9.2962  -1.3686
S    0.4316   9.5200  -2.1662
C   -0.1029  11.2581  -2.4594
S   -1.9260  11.1637  -2.7992
S   -5.1130   7.4816   0.0554
C   -4.3257   6.5774   1.4729
S   -2.6674   6.0381   0.8636
C   -4.1706   7.5109   2.6691
C   -5.1636   5.3515   1.8148
C    0.6094  11.8168  -3.6852
C    0.1529  12.1033  -1.2156
C   -2.1419  -9.7379   1.7131
C   -1.0368  -9.0211   1.2411
C   -1.1978  -7.9341   0.3582
C   -2.5079  -7.5876  -0.0320
C   -3.6189  -8.3005   0.4327
C   -3.4457  -9.3835   1.3179
S   -2.8427  -6.2669  -1.1636
C   -4.4611  -6.9743  -1.7168
S   -5.1852  -7.7603  -0.2029
S   -1.7822 -11.0675   2.8263
C   -0.1537 -10.3550   3.3574
S    0.5423  -9.5495   1.8431
C   -0.3636  -9.3197   4.4565
C    0.7720 -11.4794   3.8026
C   -5.3728  -5.8495  -2.1889
C   -4.2381  -8.0230  -2.8002
C   -0.0493  -7.1912  -0.1419
C   -0.5890  -3.5725   0.8315
C   -0.5859  -4.9708   0.8846
C   -0.0838  -5.7390  -0.1932
C    0.4097  -5.0339  -1.3162
C    0.4345  -3.6356  -1.3393
C   -0.0672  -2.8821  -0.2708
S    0.8362  -5.8302  -2.8323
C    1.8527  -4.4095  -3.4545
S    0.9777  -2.8871  -2.8486
S   -1.1079  -2.7317   2.3013
C   -2.0013  -4.2024   2.9964
S   -1.0156  -5.6729   2.4473
C   -2.0033  -4.1315   4.5173
C   -3.4120  -4.2813   2.4236
C    1.8673  -4.4207  -4.9767
C    3.2572  -4.4811  -2.8659
C    3.5893  -8.1480  -0.5266
C    2.4340  -7.4600  -0.1437
C    1.1562  -7.9032  -0.5483
C    1.0845  -9.0487  -1.3667
C    2.2323  -9.7669  -1.7348
C    3.5070  -9.2992  -1.3443
S   -0.4220  -9.5200  -2.1608
C    0.1122 -11.2590  -2.4494
S    1.9369 -11.1668  -2.7804
S    5.1137  -7.4846   0.0856
C    4.3203  -6.5782   1.4982
S    2.6657  -6.0375   0.8798
C    4.1578  -7.5105   2.6944
C    5.1578  -5.3530   1.8434
C   -0.5946 -11.8184  -3.6781
C   -0.1505 -12.1024  -1.2058
C    0.0382   1.4017  -0.2947
C   -1.1855   0.7253  -0.3046
C   -1.2211  -0.6621  -0.2963
C   -0.0337  -1.4003  -0.2936
C    1.1900  -0.7239  -0.2993
C    1.2256   0.6636  -0.2921
C    4.5628  10.1967   1.8398
O    4.4659  11.3630   2.1761
O    5.7092   9.4985   1.9144
C    6.8752  10.2502   2.3237
C   -4.7664   9.8814  -1.8515
O   -5.8529   9.3405  -1.7307
O   -4.6092  11.0637  -2.4690
C   -5.8088  11.6491  -3.0232
C   -4.5720 -10.1911   1.8253
O   -4.4773 -11.3573   2.1624
O   -5.7185  -9.4924   1.8938
C   -6.8868 -10.2435   2.2976
C    4.7740  -9.8859  -1.8208
O    5.8604  -9.3461  -1.6948
O    4.6190 -11.0699  -2.4357
C    5.8209 -11.6576  -2.9822
H    0.7752   9.8043   5.3359
H    1.0160   8.5277   4.1187
H   -0.6197   8.8756   4.7236
H   -0.9383  12.2181   2.9967
H   -0.3711  11.9849   4.6695
H   -1.7638  11.0671   4.0777
H    5.5361   5.1070  -1.3877
H    6.3568   6.2672  -2.4630
H    4.9498   5.3684  -3.0495
H    5.2129   8.4607  -3.0834
H    3.5990   8.8299  -2.4280
H    3.7834   7.5632  -3.6617
H    2.5459   3.2539   4.8515
H    0.9717   4.0853   4.9202
H    2.4894   5.0205   4.9311
H    3.3884   4.3472   1.3415
H    3.9741   3.3979   2.7320
H    3.9078   5.1794   2.8213
H   -2.3353   5.3288  -5.3462
H   -2.4170   3.5613  -5.3577
H   -0.8315   4.3730  -5.3979
H   -3.2164   4.4829  -1.7892
H   -3.8285   3.6206  -3.2246
H   -3.7315   5.4027  -3.2173
H   -5.1567   7.8562   3.0004
H   -3.5612   8.3821   2.4112
H   -3.6848   6.9780   3.4946
H   -5.2661   4.6841   0.9552
H   -6.1607   5.6648   2.1428
H   -4.6974   4.8023   2.6399
H    1.6875  11.8588  -3.4957
H    0.2654  12.8386  -3.8778
H    0.4254  11.2055  -4.5722
H   -0.3592  11.6863  -0.3434
H   -0.2041  13.1261  -1.3820
H    1.2277  12.1311  -1.0020
H   -0.8002  -9.7999   5.3396
H   -1.0345  -8.5234   4.1210
H    0.5981  -8.8724   4.7333
H    0.9221 -12.2155   3.0084
H    0.3477 -11.9816   4.6787
H    1.7436 -11.0648   4.0929
H   -5.5265  -5.1012  -1.4070
H   -6.3438  -6.2606  -2.4858
H   -4.9335  -5.3630  -3.0663
H   -5.1993  -8.4549  -3.1016
H   -3.5887  -8.8259  -2.4392
H   -3.7663  -7.5589  -3.6736
H   -2.5620  -3.2481   4.8441
H   -0.9885  -4.0803   4.9196
H   -2.5067  -5.0147   4.9250
H   -3.3912  -4.3436   1.3315
H   -3.9819  -3.3931   2.7190
H   -3.9166  -5.1745   2.8098
H    2.3608  -5.3316  -5.3318
H    2.4421  -3.5641  -5.3448
H    0.8569  -4.3763  -5.3905
H    3.2277  -4.4820  -1.7722
H    3.8451  -3.6211  -3.2061
H    3.7485  -5.4032  -3.1974
H    5.1417  -7.8566   3.0312
H    3.5489  -8.3813   2.4339
H    3.6681  -6.9764   3.5168
H    5.2658  -4.6865   0.9838
H    6.1528  -5.6674   2.1770
H    4.6879  -4.8025   2.6655
H   -1.6737 -11.8593  -3.4938
H   -0.2505 -12.8407  -3.8678
H   -0.4058 -11.2082  -4.5647
H    0.3576 -11.6849  -0.3316
H    0.2063 -13.1258  -1.3692
H   -1.2265 -12.1289  -0.9975
H   -2.1109   1.2943  -0.3101
H   -2.1747  -1.1823  -0.2920
H    2.1154  -1.2929  -0.3010
H    2.1791   1.1837  -0.2844
H    7.7020   9.5414   2.2843
H    6.7383  10.6309   3.3390
H    7.0451  11.0846   1.6383
H   -5.4807  12.5706  -3.5037
H   -6.5267  11.8617  -2.2268
H   -6.2590  10.9695  -3.7514
H   -7.7133  -9.5346   2.2534
H   -6.7553 -10.6234   3.3140
H   -7.0534 -11.0785   1.6121
H    5.4944 -12.5803  -3.4618
H    6.5347 -11.8686  -2.1818
H    6.2751 -10.9804  -3.7102
```

***p*-xyl-(TAM)_2_^••^** in vacuum

```
C    1.6600   9.7502   2.1926
C    0.7376   9.0307   1.4249
C    1.1453   7.9338   0.6377
C    2.5135   7.5886   0.6427
C    3.4434   8.3069   1.4013
C    3.0217   9.3930   2.1945
S    3.1569   6.2570  -0.3321
C    4.8644   6.9623  -0.3988
S    5.1268   7.7679   1.2473
S    1.0032  11.0904   3.1441
C   -0.7154  10.3959   3.1698
S   -0.9453   9.5715   1.5310
C   -0.8589   9.3762   4.2949
C   -1.7172  11.5359   3.3043
C    4.9667   7.9941  -1.5171
C    5.8716   5.8325  -0.5723
C    0.1910   7.1742  -0.1535
C    0.4207   3.5618   0.9528
C    0.4370   4.9606   0.9950
C    0.2519   5.7221  -0.1841
C    0.0753   5.0120  -1.3950
C    0.0195   3.6156  -1.4095
C    0.1842   2.8708  -0.2395
S    0.0746   5.7882  -2.9791
C   -0.8048   4.3843  -3.8116
S   -0.1603   2.8461  -2.9903
S    0.5378   2.7157   2.5013
C    1.2408   4.1811   3.3983
S    0.4598   5.6668   2.6140
C    0.8457   4.1202   4.8680
C    2.7535   4.2345   3.2126
C   -0.4399   4.3644  -5.2898
C   -2.3080   4.5116  -3.5921
C   -3.2320   8.0505  -1.4968
C   -2.2058   7.3876  -0.8180
C   -0.8766   7.8595  -0.8708
C   -0.6127   9.0033  -1.6503
C   -1.6355   9.6969  -2.3139
C   -2.9584   9.2036  -2.2693
S    1.0417   9.4996  -2.0204
C    0.5714  11.2233  -2.4638
S   -1.1028  11.0997  -3.2551
S   -4.8514   7.3592  -1.3089
C   -4.4371   6.4709   0.2675
S   -2.6710   5.9595   0.1146
C   -4.6090   7.4111   1.4572
C   -5.3140   5.2305   0.3903
C    1.5636  11.7791  -3.4792
C    0.4951  12.0890  -1.2097
C   -1.6588  -9.7468   2.1913
C   -0.7365  -9.0273   1.4234
C   -1.1442  -7.9303   0.6364
C   -2.5124  -7.5850   0.6416
C   -3.4422  -8.3032   1.4003
C   -3.0204  -9.3894   2.1934
S   -3.1557  -6.2533  -0.3330
C   -4.8633  -6.9586  -0.3995
S   -5.1256  -7.7642   1.2465
S   -1.0019 -11.0869   3.1428
C    0.7166 -10.3924   3.1683
S    0.9464  -9.5683   1.5293
C    0.8601  -9.3724   4.2931
C    1.7186 -11.5323   3.3030
C   -4.9658  -7.9903  -1.5179
C   -5.8705  -5.8287  -0.5729
C   -0.1900  -7.1707  -0.1548
C   -0.4197  -3.5585   0.9522
C   -0.4358  -4.9573   0.9941
C   -0.2508  -5.7185  -0.1851
C   -0.0743  -5.0082  -1.3959
C   -0.0187  -3.6117  -1.4101
C   -0.1835  -2.8672  -0.2401
S   -0.0739  -5.7840  -2.9802
C    0.8052  -4.3799  -3.8125
S    0.1606  -2.8419  -2.9909
S   -0.5369  -2.7127   2.5008
C   -1.2397  -4.1783   3.3975
S   -0.4585  -5.6637   2.6131
C   -0.8447  -4.1176   4.8673
C   -2.7524  -4.2319   3.2118
C    0.4400  -4.3598  -5.2907
C    2.3085  -4.5070  -3.5934
C    3.2329  -8.0469  -1.4986
C    2.2068  -7.3840  -0.8195
C    0.8776  -7.8559  -0.8724
C    0.6135  -8.9994  -1.6521
C    1.6362  -9.6930  -2.3158
C    2.9591  -9.1998  -2.2713
S   -1.0410  -9.4955  -2.0223
C   -0.5709 -11.2191  -2.4662
S    1.1032 -11.0954  -3.2575
S    4.8523  -7.3556  -1.3109
C    4.4382  -6.4673   0.2656
S    2.6721  -5.9560   0.1131
C    4.6105  -7.4074   1.4553
C    5.3151  -5.2269   0.3882
C   -1.5632 -11.7744  -3.4818
C   -0.4947 -12.0851  -1.2124
C    0.0765   1.3902  -0.2525
C   -1.1878   0.7801  -0.2491
C   -1.2348  -0.6114  -0.2458
C   -0.0758  -1.3866  -0.2527
C    1.1885  -0.7765  -0.2496
C    1.2355   0.6150  -0.2460
C   -2.4468   1.6009  -0.2429
C    2.4475  -1.5973  -0.2439
C    3.9483  10.2023   3.0071
O    3.7527  11.3600   3.3214
O    5.0411   9.5087   3.3899
C    6.0269  10.2679   4.1176
C   -4.0686   9.7633  -3.0609
O   -5.1396   9.2065  -3.2197
O   -3.7801  10.9529  -3.6256
C   -4.8150  11.5061  -4.4616
C   -3.9470 -10.1987   3.0062
O   -3.7515 -11.3564   3.3203
O   -5.0395  -9.5050   3.3894
C   -6.0253 -10.2641   4.1173
C    4.0692  -9.7594  -3.0631
O    5.1401  -9.2025  -3.2222
O    3.7806 -10.9490  -3.6278
C    4.8154 -11.5020  -4.4641
H   -0.6912   9.8618   5.2631
H   -0.1341   8.5652   4.1790
H   -1.8659   8.9432   4.2806
H   -1.6132  12.2574   2.4898
H   -1.5664  12.0541   4.2578
H   -2.7386  11.1395   3.2995
H    4.7705   7.5164  -2.4837
H    5.9714   8.4322  -1.5310
H    4.2362   8.7957  -1.3749
H    5.7909   5.0989   0.2340
H    6.8894   6.2377  -0.5839
H    5.7036   5.3275  -1.5298
H    1.2826   3.2301   5.3335
H   -0.2397   4.0874   4.9891
H    1.2385   4.9970   5.3942
H    3.0156   4.2982   2.1526
H    3.2136   3.3358   3.6395
H    3.1589   5.1204   3.7151
H   -0.7928   5.2834  -5.7701
H   -0.9344   3.5203  -5.7826
H    0.6396   4.2774  -5.4347
H   -2.5495   4.5442  -2.5254
H   -2.8247   3.6590  -4.0481
H   -2.6748   5.4385  -4.0482
H   -5.6524   7.7402   1.5262
H   -3.9694   8.2914   1.3475
H   -4.3319   6.8965   2.3846
H   -5.1775   4.5598  -0.4622
H   -6.3683   5.5236   0.4448
H   -5.0713   4.6883   1.3110
H    2.5590  11.8444  -3.0263
H    1.2674  12.7919  -3.7733
H    1.6177  11.1515  -4.3721
H   -0.2097  11.6712  -0.4853
H    0.1717  13.1021  -1.4753
H    1.4804  12.1420  -0.7320
H    0.6925  -9.8577   5.2615
H    0.1353  -8.5615   4.1771
H    1.8672  -8.9393   4.2788
H    1.6145 -12.2539   2.4886
H    1.5678 -12.0503   4.2566
H    2.7400 -11.1358   3.2980
H   -4.7696  -7.5126  -2.4845
H   -5.9704  -8.4284  -1.5317
H   -4.2353  -8.7919  -1.3757
H   -5.7897  -5.0951   0.2333
H   -6.8883  -6.2338  -0.5845
H   -5.7026  -5.3237  -1.5305
H   -1.2817  -3.2277   5.3329
H    0.2407  -4.0846   4.9885
H   -1.2374  -4.9946   5.3933
H   -3.0145  -4.2954   2.1518
H   -3.2126  -3.3334   3.6389
H   -3.1577  -5.1180   3.7141
H    0.7928  -5.2786  -5.7713
H    0.9342  -3.5156  -5.7834
H   -0.6396  -4.2729  -5.4353
H    2.5502  -4.5399  -2.5267
H    2.8249  -3.6543  -4.0493
H    2.6753  -5.4338  -4.0498
H    5.6539  -7.7366   1.5240
H    3.9709  -8.2878   1.3457
H    4.3335  -6.8928   2.3827
H    5.1784  -4.5562  -0.4643
H    6.3694  -5.5199   0.4425
H    5.0726  -4.6846   1.3090
H   -2.5586 -11.8398  -3.0288
H   -1.2671 -12.7871  -3.7763
H   -1.6174 -11.1465  -4.3744
H    0.2102 -11.6677  -0.4879
H   -0.1714 -13.0982  -1.4784
H   -1.4800 -12.1382  -0.7347
H   -2.1986  -1.1153  -0.2314
H    2.1993   1.1189  -0.2318
H   -2.4202   2.3602   0.5467
H   -3.3243   0.9675  -0.0895
H   -2.5674   2.1340  -1.1941
H    2.4212  -2.3567   0.5456
H    3.3251  -0.9640  -0.0908
H    2.5678  -2.1303  -1.1953
H    6.8430   9.5714   4.3109
H    5.6020  10.6394   5.0542
H    6.3725  11.1153   3.5191
H   -4.4050  12.4407  -4.8453
H   -5.7190  11.6902  -3.8747
H   -5.0515  10.8194  -5.2791
H   -6.8412  -9.5674   4.3109
H   -5.6002 -10.6358   5.0536
H   -6.3712 -11.1113   3.5187
H    4.4054 -12.4367  -4.8478
H    5.7195 -11.6860  -3.8775
H    5.0515 -10.8153  -5.2817
```

***p*-xyl-(TAM)_2_^••^** in CPCM(Toluene)

```
C    1.6389   9.7814   2.1892
C    0.7212   9.0592   1.4180
C    1.1310   7.9481   0.6518
C    2.4978   7.5971   0.6742
C    3.4223   8.3174   1.4380
C    2.9975   9.4143   2.2146
S    3.1483   6.2546  -0.2805
C    4.8636   6.9476  -0.3331
S    5.1037   7.7648   1.3124
S    0.9743  11.1366   3.1152
C   -0.7503  10.4533   3.1313
S   -0.9590   9.6126   1.4958
C   -0.9100   9.4463   4.2646
C   -1.7477  11.5990   3.2411
C    4.9878   7.9705  -1.4563
C    5.8653   5.8102  -0.4829
C    0.1790   7.1782  -0.1327
C    0.3813   3.5853   1.0446
C    0.4039   4.9848   1.0600
C    0.2335   5.7246  -0.1352
C    0.0682   4.9919  -1.3345
C    0.0193   3.5951  -1.3241
C    0.1665   2.8720  -0.1383
S    0.0799   5.7362  -2.9350
C   -0.7784   4.3098  -3.7552
S   -0.1287   2.7951  -2.8938
S    0.4652   2.7695   2.6118
C    1.1731   4.2422   3.4948
S    0.4198   5.7213   2.6663
C    0.7561   4.2154   4.9588
C    2.6883   4.2735   3.3299
C   -0.3976   4.2666  -5.2282
C   -2.2843   4.4265  -3.5526
C   -3.2177   8.0230  -1.5610
C   -2.2086   7.3907  -0.8296
C   -0.8760   7.8529  -0.8796
C   -0.5931   8.9659  -1.6957
C   -1.5991   9.6268  -2.4169
C   -2.9236   9.1357  -2.3834
S    1.0705   9.4474  -2.0447
C    0.6156  11.1462  -2.5945
S   -1.0397  10.9780  -3.4173
S   -4.8415   7.3397  -1.3737
C   -4.4685   6.5293   0.2552
S   -2.6985   6.0107   0.1609
C   -4.6662   7.5263   1.3924
C   -5.3494   5.2967   0.4174
C    1.6338  11.6461  -3.6125
C    0.5081  12.0785  -1.3920
C   -1.6391  -9.7774   2.1875
C   -0.7210  -9.0555   1.4164
C   -1.1302  -7.9441   0.6505
C   -2.4968  -7.5925   0.6728
C   -3.4217  -8.3124   1.4364
C   -2.9975  -9.4096   2.2130
S   -3.1467  -6.2495  -0.2819
C   -4.8623  -6.9416  -0.3344
S   -5.1028  -7.7588   1.3110
S   -0.9753 -11.1331   3.1132
C    0.7497 -10.4507   3.1296
S    0.9589  -9.6098   1.4942
C    0.9098  -9.4440   4.2631
C    1.7466 -11.5969   3.2392
C   -4.9871  -7.9644  -1.4578
C   -5.8635  -5.8037  -0.4841
C   -0.1778  -7.1745  -0.1339
C   -0.3784  -3.5817   1.0441
C   -0.4014  -4.9812   1.0593
C   -0.2316  -5.7208  -0.1362
C   -0.0662  -4.9881  -1.3353
C   -0.0169  -3.5912  -1.3247
C   -0.1636  -2.8682  -0.1388
S   -0.0785  -5.7321  -2.9360
C    0.7800  -4.3057  -3.7561
S    0.1309  -2.7910  -2.8944
S   -0.4616  -2.7662   2.6114
C   -1.1696  -4.2388   3.4945
S   -0.4171  -5.7179   2.6655
C   -0.7522  -4.2123   4.9583
C   -2.6849  -4.2696   3.3300
C    0.3989  -4.2621  -5.2290
C    2.2859  -4.4227  -3.5538
C    3.2181  -8.0205  -1.5631
C    2.2095  -7.3879  -0.8313
C    0.8767  -7.8496  -0.8810
C    0.5932  -8.9624  -1.6972
C    1.5988  -9.6236  -2.4187
C    2.9235  -9.1331  -2.3855
S   -1.0707  -9.4432  -2.0458
C   -0.6166 -11.1421  -2.5959
S    1.0385 -10.9745  -3.4192
S    4.8423  -7.3379  -1.3762
C    4.4700  -6.5273   0.2527
S    2.7001  -6.0081   0.1589
C    4.6677  -7.5242   1.3900
C    5.3513  -5.2949   0.4146
C   -1.6353 -11.6416  -3.6136
C   -0.5091 -12.0746  -1.3934
C    0.0682   1.3904  -0.1335
C   -1.1927   0.7725  -0.1292
C   -1.2307  -0.6201  -0.1259
C   -0.0653  -1.3867  -0.1337
C    1.1955  -0.7688  -0.1295
C    1.2336   0.6238  -0.1259
C   -2.4576   1.5843  -0.1291
C    2.4605  -1.5806  -0.1293
C    3.9237  10.2217   3.0323
O    3.7438  11.3930   3.3122
O    4.9858   9.5147   3.4563
C    5.9813  10.2605   4.1943
C   -4.0103   9.6556  -3.2351
O   -5.0734   9.0815  -3.4017
O   -3.7105  10.8172  -3.8389
C   -4.7145  11.3351  -4.7405
C   -3.9242 -10.2166   3.0304
O   -3.7448 -11.3880   3.3102
O   -4.9859  -9.5092   3.4546
C   -5.9818 -10.2545   4.1925
C    4.0096  -9.6533  -3.2376
O    5.0730  -9.0796  -3.4044
O    3.7092 -10.8147  -3.8415
C    4.7128 -11.3329  -4.7435
H   -0.7569   9.9448   5.2286
H   -0.1853   8.6322   4.1700
H   -1.9192   9.0189   4.2410
H   -1.6318  12.3128   2.4213
H   -1.6053  12.1232   4.1923
H   -2.7692  11.2034   3.2276
H    4.8047   7.4841  -2.4211
H    5.9970   8.3979  -1.4589
H    4.2625   8.7800  -1.3319
H    5.7695   5.0815   0.3264
H    6.8844   6.2118  -0.4830
H    5.7065   5.3021  -1.4402
H    1.1790   3.3302   5.4457
H   -0.3311   4.1966   5.0671
H    1.1524   5.0992   5.4702
H    2.9687   4.3046   2.2729
H    3.1291   3.3814   3.7891
H    3.0942   5.1660   3.8201
H   -0.7572   5.1729  -5.7269
H   -0.8794   3.4076  -5.7073
H    0.6841   4.1891  -5.3623
H   -2.5383   4.4672  -2.4889
H   -2.7866   3.5641  -4.0056
H   -2.6509   5.3439  -4.0278
H   -5.7120   7.8530   1.4222
H   -4.0271   8.4038   1.2569
H   -4.4125   7.0546   2.3487
H   -5.1957   4.5841  -0.3976
H   -6.4033   5.5952   0.4351
H   -5.1262   4.8024   1.3692
H    2.6163  11.7343  -3.1362
H    1.3448  12.6416  -3.9660
H    1.7110  10.9723  -4.4693
H   -0.2195  11.7050  -0.6655
H    0.1987  13.0764  -1.7232
H    1.4824  12.1550  -0.8955
H    0.7564  -9.9427   5.2269
H    0.1856  -8.6296   4.1685
H    1.9192  -9.0171   4.2396
H    1.6303 -12.3105   2.4192
H    1.6038 -12.1213   4.1903
H    2.7682 -11.2018   3.2258
H   -4.8038  -7.4780  -2.4225
H   -5.9965  -8.3912  -1.4604
H   -4.2623  -8.7743  -1.3334
H   -5.7673  -5.0751   0.3252
H   -6.8828  -6.2047  -0.4842
H   -5.7044  -5.2956  -1.4413
H   -1.1747  -3.3270   5.4454
H    0.3351  -4.1938   5.0663
H   -1.1485  -5.0961   5.4696
H   -2.9656  -4.3003   2.2731
H   -3.1252  -3.3774   3.7895
H   -3.0909  -5.1620   3.8201
H    0.7582  -5.1685  -5.7279
H    0.8807  -3.4033  -5.7081
H   -0.6829  -4.1844  -5.3629
H    2.5402  -4.4636  -2.4902
H    2.7883  -3.5604  -4.0069
H    2.6522  -5.3402  -4.0292
H    5.7135  -7.8512   1.4195
H    4.0283  -8.4015   1.2548
H    4.4145  -7.0523   2.3464
H    5.1976  -4.5824  -0.4004
H    6.4051  -5.5937   0.4320
H    5.1286  -4.8005   1.3665
H   -2.6177 -11.7294  -3.1371
H   -1.3468 -12.6372  -3.9673
H   -1.7124 -10.9677  -4.4704
H    0.2188 -11.7014  -0.6672
H   -0.2002 -13.0726  -1.7249
H   -1.4834 -12.1508  -0.8967
H   -2.1920  -1.1292  -0.1156
H    2.1949   1.1329  -0.1157
H   -2.4400   2.3446   0.6599
H   -3.3310   0.9446   0.0203
H   -2.5783   2.1159  -1.0815
H    2.4428  -2.3411   0.6596
H    3.3339  -0.9410   0.0203
H    2.5813  -2.1120  -1.0818
H    6.7730   9.5442   4.4129
H    5.5472  10.6530   5.1174
H    6.3608  11.0862   3.5869
H   -4.2843  12.2477  -5.1528
H   -5.6352  11.5535  -4.1934
H   -4.9210  10.6101  -5.5320
H   -6.7730  -9.5378   4.4113
H   -5.5478 -10.6474   5.1155
H   -6.3617 -11.0799   3.5849
H    4.2820 -12.2452  -5.1558
H    5.6335 -11.5518  -4.1966
H    4.9194 -10.6078  -5.5349
```

## **6.4 DFT calculation of *J* on representative conformers from MD**

From the molecular dynamics trajectory shown in Figure S12, we picked distinct conformers characterized by the C-C distance r_C-C_, the dihedral angles $\theta_{1}$ and $\theta_{2}$, and the opening angle $\varphi$. To do so, we did a simultaneous binning across r_C-C_, $\theta_{1}$ and $\theta_{2}$, and $\varphi$. After the binning, we determined the 14 most-populated bins and calculated the exchange-coupling constant *J* of these conformers on different levels of density functional theory (see Table S4). The conformers are depicted graphically below along with the corresponding geometric parameters r_C-C_, $\theta_{1}$, $\theta_{2}$, and $\varphi$.

**Conformer 1:**

|  |  |  |
| --- | --- | --- |
|  |  |  |
|  | r_C-C_/ Å | 13.0 |
|  | r_spin-spin_ / Å | 12.2 |
|  | θ_1_ / ° | 60 |
|  | θ_2_ / ° | 100 |
|  | $\varphi$ / ° | 135 |
|  |  |  |
|  |  |  |
|  |  |  |
|  |  |  |

**Conformer 2:**

|  |  |  |
| --- | --- | --- |
|  |  |  |
|  | r_C-C_/ Å | 12.9 |
|  | r_spin-spin_ / Å | 12.2 |
|  | θ_1_ / ° | 120 |
|  | θ_2_ / ° | 115 |
|  | $\varphi$ / ° | 130 |
|  |  |  |
|  |  |  |
|  |  |  |
|  |  |  |

**Conformer 3:**

|  |  |  |
| --- | --- | --- |
|  |  |  |
|  | r_C-C_/ Å | 12.9 |
|  | r_spin-spin_ / Å | 12.3 |
|  | θ_1_ / ° | 119 |
|  | θ_2_ / ° | 121 |
|  | $\varphi$ / ° | 131 |
|  |  |  |
|  |  |  |
|  |  |  |
|  |  |  |

**Conformer 4:**

|  |  |  |
| --- | --- | --- |
|  |  |  |
|  | r_C-C_/ Å | 13.1 |
|  | r_spin-spin_ / Å | 12.3 |
|  | θ_1_ / ° | 126 |
|  | θ_2_ / ° | 67 |
|  | $\varphi$ / ° | 132 |
|  |  |  |
|  |  |  |
|  |  |  |
|  |  |  |

**Conformer 5:**

|  |  |  |
| --- | --- | --- |
|  |  |  |
|  | r_C-C_/ Å | 12.7 |
|  | r_spin-spin_ / Å | 12.1 |
|  | θ_1_ / ° | 52 |
|  | θ_2_ / ° | 109 |
|  | $\varphi$ / ° | 125 |
|  |  |  |
|  |  |  |
|  |  |  |
|  |  |  |

**Conformer 6:**

|  |  |  |
| --- | --- | --- |
|  |  |  |
|  | r_C-C_/ Å | 12.9 |
|  | r_spin-spin_ / Å | 12.2 |
|  | θ_1_ / ° | 63 |
|  | θ_2_ / ° | 121 |
|  | $\varphi$ / ° | 131 |
|  |  |  |
|  |  |  |
|  |  |  |
|  |  |  |

**Conformer 7:**

|  |  |  |
| --- | --- | --- |
|  |  |  |
|  | r_C-C_/ Å | 13.0 |
|  | r_spin-spin_ / Å | 12.3 |
|  | θ_1_ / ° | 78 |
|  | θ_2_ / ° | 119 |
|  | $\varphi$ / ° | 132 |
|  |  |  |
|  |  |  |
|  |  |  |
|  |  |  |

**Conformer 8:**

|  |  |  |
| --- | --- | --- |
|  |  |  |
|  | r_C-C_/ Å | 12.9 |
|  | r_spin-spin_ / Å | 12.2 |
|  | θ_1_ / ° | 116 |
|  | θ_2_ / ° | 99 |
|  | $\varphi$ / ° | 132 |
|  |  |  |
|  |  |  |
|  |  |  |
|  |  |  |

**Conformer 9:**

|  |  |  |
| --- | --- | --- |
|  |  |  |
|  | r_C-C_/ Å | 12.9 |
|  | r_spin-spin_ / Å | 12.2 |
|  | θ_1_ / ° | 84 |
|  | θ_2_ / ° | 131 |
|  | $\varphi$ / ° | 130 |
|  |  |  |
|  |  |  |
|  |  |  |
|  |  |  |

**Conformer 10:**

|  |  |  |
| --- | --- | --- |
|  |  |  |
|  | r_C-C_/ Å | 13.0 |
|  | r_spin-spin_ / Å | 12.3 |
|  | θ_1_ / ° | 90 |
|  | θ_2_ / ° | 132 |
|  | $\varphi$ / ° | 133 |
|  |  |  |
|  |  |  |
|  |  |  |
|  |  |  |

**Conformer 11:**

|  |  |  |
| --- | --- | --- |
|  |  |  |
|  | r_C-C_/ Å | 12.9 |
|  | r_spin-spin_ / Å | 12.1 |
|  | θ_1_ / ° | 68 |
|  | θ_2_ / ° | 120 |
|  | $\varphi$ / ° | 128 |
|  |  |  |
|  |  |  |
|  |  |  |
|  |  |  |

**Conformer 12:**

|  |  |  |
| --- | --- | --- |
|  |  |  |
|  | r_C-C_/ Å | 13.0 |
|  | r_spin-spin_ / Å | 12.3 |
|  | θ_1_ / ° | 119 |
|  | θ_2_ / ° | 60 |
|  | $\varphi$ / ° | 131 |
|  |  |  |
|  |  |  |
|  |  |  |
|  |  |  |

**Conformer 13:**

|  |  |  |
| --- | --- | --- |
|  |  |  |
|  | r_C-C_/ Å | 12.6 |
|  | r_spin-spin_ / Å | 12.0 |
|  | θ_1_ / ° | 74 |
|  | θ_2_ / ° | 126 |
|  | $\varphi$ / ° | 124 |
|  |  |  |
|  |  |  |
|  |  |  |
|  |  |  |

**Conformer 14:**

|  |  |  |
| --- | --- | --- |
|  |  |  |
|  | r_C-C_/ Å | 13.1 |
|  | r_spin-spin_ / Å | 12.4 |
|  | θ_1_ / ° | 63 |
|  | θ_2_ / ° | 60 |
|  | $\varphi$ / ° | 131 |
|  |  |  |
|  |  |  |
|  |  |  |
|  |  |  |

**Table S4:** Calculated J-values (in GHz) of the 14 conformers of **m-ben-(TAM)_2_^••^** from the binning of the MD trajectory using functionals (FUNC) on the FUNC/def2-TZVPP//r^2^SCAN-3c/def2-mTZVPP level of theory.

| **Conf.** | **r_C-C_**  **/ Å** | **r_spin-spin_**  **/ Å** | **θ_1_**  **/ °** | **θ_2_**  **/ °** | $\varphi$  **/ °** | **J** | | | | | | | | |
| --- | --- | --- | --- | --- | --- | --- | --- | --- | --- | --- | --- | --- | --- | --- |
|  |  |  |  |  |  | **B3LYP** | **M06L** | **PBE0** | **r2scan3c** | **TPSS** | **TPSSh** | **wB97XD4** | **wB97XV** |  |
| Conf. 1 | 13.0 | 12.2 | 60 | 100 | 135 | -1.029 cm^-1^  -30.85 GHz | -1.657 cm^-1^  -49.68 GHz | -0.540 cm^-1^  -16.19 GHz | -1.471 cm^-1^  -44.10 GHz | -2.718 cm^-1^  -81.48 GHz | -1.388 cm^-1^  -41.61 GHz | -0.042 cm^-1^  1.259 GHz | -0.160 cm^-1^  -4.797 GHz |  |
| Conf. 2 | 12.9 | 12.2 | 120 | 115 | 130 | 1.447 cm^-1^  43.38 GHz | 0.042 cm^-1^  1.259 GHz | 2.892 cm^-1^  86.70 GHz | 1.931 cm^-1^  57.89 GHz | -0.423 cm^-1^  -12.68 GHz | 1.164 cm^-1^  34.90 GHz | 4.074 cm^-1^  122.1 GHz | 4.097 cm^-1^  122.8 GHz |  |
| Conf. 3 | 12.9 | 12.3 | 119 | 121 | 131 | 4.326 cm^-1^  129.7 GHz | 2.397 cm^-1^  71.86 GHz | 4.407 cm^-1^  132.1 GHz | 4.820 cm^-1^  144.5 GHz | 2.160 cm^-1^  64.76 GHz | 3.376 cm^-1^  101.2 GHz | 5.732 cm^-1^  171.8 GHz | 5.768 cm^-1^  172.9 GHz |  |
| Conf. 4 | 13.1 | 12.3 | 126 | 67 | 132 | 0.776 cm^-1^  23.26 GHz | 0.582 cm^-1^  17.45 GHz | 0.301 cm^-1^  9.024 GHz | 1.309 cm^-1^  39.24 GHz | 0.456 cm^-1^  13.67 GHz | 0.722 cm^-1^  21.65 GHz | 8.618 cm^-1^  258.4 GHz | 142.065 cm^-1^  4259 GHz |  |
| Conf. 5 | 12.7 | 12.1 | 52 | 109 | 125 | 2.694 cm^-1^  80.76 GHz | 1.801 cm^-1^  53.99 GHz | 3.258 cm^-1^  67.67 GHz | 4.111 cm^-1^  123.25 GHz | 1.150 cm^-1^  34.48 GHz | 2.255 cm^-1^  67.60 GHz | 3.790 cm^-1^  113.6 GHz | 3.647 cm^-1^  109.3 GHz |  |
| Conf. 6 | 12.9 | 12.2 | 63 | 121 | 131 | 0.587 cm^-1^  17.60 GHz | -1.087 cm^-1^  -32.59 GHz | 1.868 cm^-1^  56.00 GHz | 1.230 cm^-1^  36.87 GHz | -3.599 cm^-1^  -107.9 GHz | -0.267 cm^-1^  -8.00 GHz | 3.202 cm^-1^  95.99 GHz | 2.989 cm^-1^  89.61 GHz |  |
| Conf. 7 | 13.0 | 12.3 | 78 | 119 | 132 | 0.299 cm^-1^  8.964 GHz | -0.572 cm^-1^  -17.15 GHz | 0.661 cm^-1^  19.82 GHz | 0.356 cm^-1^  10.67 GHz | -1.211 cm^-1^  -36.30 GHz | -0.158 cm^-1^  -4.737 GHz | 0.998 cm^-1^  29.92 GHz | 0.986 cm^-1^  29.56 GHz |  |
| Conf. 8 | 12.9 | 12.2 | 116 | 99 | 132 | 1.688 cm^-1^  50.01 GHz | 1.110 cm^-1^  33.28 GHz | -1.102 cm^-1^  -33.04 GHz | 2.150 cm^-1^  64.46 GHz | 1.103 cm^-1^  33.07 GHz | 1.187 cm^-1^  35.59 GHz | 1.849 cm^-1^  55.43 GHz | 1.872 cm^-1^  56.12 GHz |  |
| Conf. 9 | 12.9 | 12.2 | 84 | 131 | 130 | -2.231 cm^-1^  -66.88 GHz | -5.179 cm^-1^  -155.3 GHz | -1.805 cm^-1^  -55.11 GHz | -4.817 cm^-1^  -144.4 GHz | -7.777 cm^-1^  -233.1 GHz | -3.709 cm^-1^  -111.2 GHz | -0.628 cm^-1^  -18.83 GHz | -0.692 cm^-1^  -20.75 GHz |  |
| Conf. 10 | 13.0 | 12.3 | 90 | 132 | 133 | -0.018 cm^-1^  -0.540 GHz | -0.422 cm^-1^  -12.65 GHz | 0.234 cm^-1^  -7.015 GHz | -0.034 cm^-1^  -1.019 GHz | -0.826 cm^-1^  -24.76 GHz | -0.252 cm^-1^  -7.555 GHz | -1308 cm^-1^  -39213 GHz | -1157 cm^-1^  -34686 GHz |  |
| Conf. 11 | 12.9 | 12.1 | 68 | 120 | 128 | 1.427 cm^-1^  42.78 GHz | 0.856 cm^-1^  25.66 GHz | 1.578 cm^-1^  47.31 GHz | 2.148 cm^-1^  64.40 GHz | 0.492 cm^-1^  14.75 GHz | 1.046 cm^-1^  31.36 GHz | 1.935 cm^-1^  58.01 GHz | 1.943 cm^-1^  58.25 GHz |  |
| Conf. 12 | 13.0 | 12.3 | 119 | 60 | 131 | 1.578 cm^-1^  47.31 GHz | -0.532 cm^-1^  -15.95 GHz | 3.578 cm^-1^  107.3 GHz | 3.642 cm^-1^  109.2 GHz | -3.237 cm^-1^  -97.04 GHz | 0.990 cm^-1^  29.68 GHz | 6.090 cm^-1^  182.6 GHz | 6.318 cm^-1^  189.4 GHz |  |
| Conf. 13 | 12.6 | 12.0 | 74 | 126 | 124 | -0.468 cm^-1^  -14.03 GHz | 0.095 cm^-1^  2.848 GHz | 0.564 cm^-1^  16.91 GHz | 0.507 cm^-1^  15.20 GHz | -0.159 cm^-1^  -4.77 GHz | -0.043 cm^-1^  -1.29 GHz | 0.654 cm^-1^  19.61 GHz | 0.649 cm^-1^  19.46 GHz |  |
| Conf. 14 | 13.1 | 12.4 | 63 | 60 | 131 | 1.359 cm^-1^  40.74 GHz | 0.973 cm^-1^  29.17 GHz | 1.877 cm^-1^  56.27 GHz | 1.876 cm^-1^  56.24 GHz | 0.885 cm^-1^  26.53 GHz | 1.279 cm^-1^  38.34 GHz | -679 cm^-1^  -20356 GHz | -560 cm^-1^  -16788 GHz |  |

The exchange coupling constant *J* is based on the Hamiltonian -2 JS_1_S_2_, i.e., J values < 0 correspond to antiferromagnetic exchange coupling and J values > 0 correspond to ferromagnetic exchange coupling.

# **7. References**

[1]	O. Burghaus, M. Rohrer, T. Gotzinger, M. Plato, K. Mobius, “A novel high-field/high-frequency EPR and ENDOR spectrometer operating at 3 mm wavelength” *Meas. Sci. Technol.* **1992**, *3*, 765–774.

[2]	K. L. Kopp, A. Pellegrini, S. Grimme, O. Schiemann, **2026**, DOI: 10.26434/chemrxiv.10002037/v2.

[3]	F. Neese, F. Wennmohs, U. Becker, C. Riplinger, “The ORCA quantum chemistry program package” *J. Chem. Phys.* **2020**, *152*, 224108.

[4]	F. Neese, “The ORCA program system” *WIREs Comput. Mol. Sci.****.* 2012**, *2*, 73–78.

[5]	F. Neese, “Software Update: The ORCA Program System—Version 6.0***”*** *WIREs Comput. Mol. Sci.****.*** **2025**, *15*, e70019.

[6]	F. Neese, “The SHARK integral generation and digestion system” *J. Comput. Chem.* **2023**, *44*, 381–396.

[7]	F. Neese, “An improvement of the resolution of the identity approximation for the formation of the Coulomb matrix” *J. Comput. Chem.* **2003**, *24*, 1740–1747.

[8]	F. Neese, F. Wennmohs, A. Hansen, U. Becker, “Efficient, approximate and parallel Hartree–Fock and hybrid DFT calculations. A ‘chain-of-spheres’ algorithm for the Hartree–Fock exchange” *Chem. Phys.* **2009**, *356*, 98–109.

[9]	B. Helmich-Paris, B. de Souza, F. Neese, R. Izsák, “An improved chain of spheres for exchange algorithm” *J. Chem. Phys.* **2021**, *155*, 104109.

[10]	C. Bannwarth, S. Ehlert, S. Grimme, “GFN2-xTB—An Accurate and Broadly Parametrized Self-Consistent Tight-Binding Quantum Chemical Method with Multipole Electrostatics and Density-Dependent Dispersion Contributions” *J. Chem. Theory Comput.* **2019**, *15*, 1652–1671.

[11]	S. Grimme, A. Hansen, S. Ehlert, J.-M. Mewes, “r2SCAN-3c: A ‘Swiss army knife’ composite electronic-structure method” *J. Chem. Phys.* **2021**, *154*, 064103.

[12]	H. Kruse, S. Grimme, “A geometrical correction for the inter- and intra-molecular basis set superposition error in Hartree-Fock and density functional theory calculations for large systems” *J. Chem. Phys.* **2012**, *136*, 154101.

[13]	E. Caldeweyher, J.-M. Mewes, S. Ehlert, S. Grimme, “Extension and evaluation of the D4 London-dispersion model for periodic systems” *Phys. Chem. Chem. Phys.* **2020**, *22*, 8499–8512.

[14]	L. Wittmann, I. Gordiy, M. Friede, B. Helmich-Paris, S. Grimme, A. Hansen, M. Bursch, “Extension of the D3 and D4 London dispersion corrections to the full actinides series” *Phys. Chem. Chem. Phys.* **2024**, *26*, 21379–21394.

[15]	Pettersen, E. F.; Goddard, T. D.; Huang, C. C.; Couch, G. S.; Greenblatt, D. M.; Meng, E. C.; Ferrin, T. E. UCSF ChimeraX: A Visualization System for Exploratory Research and Analysis. *J. Comput. Chem.* **2004**, *25* (13), 1605–1612.

[16]	Y. Zhao, D. G. Truhlar, “The M06 suite of density functionals for main group thermochemistry, thermochemical kinetics, noncovalent interactions, excited states, and transition elements: two new functionals and systematic testing of four M06-class functionals and 12 other functionals” *Theor. Chem. Acc.* **2008**, *120*, 215–241.

[17]	J. Tao, J. P. Perdew, V. N. Staroverov, G. E. Scuseria, “Climbing the Density Functional Ladder: Nonempirical Meta–Generalized Gradient Approximation Designed for Molecules and Solids” *Phys. Rev. Lett.* **2003**, *91*, 146401.

[18]	V. N. Staroverov, G. E. Scuseria, J. Tao, J. P. Perdew, “Erratum: ‘Comparative assessment of a new nonempirical density functional: Molecules and hydrogen-bonded complexes’ [J. Chem. Phys. **119** , 12129 (2003)]” *J. Chem. Phys.* **2004**, *121*, 11507–11507.

[19]	A. D. Becke, “Density-functional thermochemistry. III. The role of exact exchange” *J. Chem. Phys.* **1993**, *98*, 5648–5652.

[20]	J. P. Perdew, M. Ernzerhof, K. Burke, “Rationale for mixing exact exchange with density functional approximations” *J. Chem. Phys.* **1996**, *105*, 9982–9985.

[21]	C. Adamo, V. Barone, “Toward reliable density functional methods without adjustable parameters: The PBE0 model” *J. Chem. Phys.* **1999**, *110*, 6158–6170.

[22]	M. Ernzerhof, G. E. Scuseria, “Assessment of the Perdew–Burke–Ernzerhof exchange-correlation functional” *J. Chem. Phys.* **1999**, *110*, 5029–5036.

[23]	J.-D. Chai, M. Head-Gordon, “Systematic optimization of long-range corrected hybrid density functionals” *J. Chem. Phys.* **2008**, *128*, 084106.

[24]	E. Caldeweyher, C. Bannwarth, S. Grimme, “Extension of the D3 dispersion coefficient model” *J. Chem. Phys.* **2017**, *147*, 034112.

[25]	E. Caldeweyher, S. Ehlert, A. Hansen, H. Neugebauer, S. Spicher, C. Bannwarth, S. Grimme, “A generally applicable atomic-charge dependent London dispersion correction” *J. Chem. Phys.* **2019**, *150*, 154122.

[26]	N. Mardirossian, M. Head-Gordon, “ωB97X-V: A 10-parameter, range-separated hybrid, generalized gradient approximation density functional with nonlocal correlation, designed by a survival-of-the-fittest strategy” *Phys. Chem. Chem. Phys.* **2014**, *16*, 9904–9924.

[27]	G. J. Martyna, M. L. Klein, M. Tuckerman, “Nosé–Hoover chains: The canonical ensemble via continuous dynamics” *J. Chem. Phys.* **1992**, *97*, 2635–2643.

[28]	G. J. Martyna, M. E. Tuckerman, D. J. Tobias, M. L. Klein, “Explicit reversible integrators for extended systems dynamics” *Mol. Phys.* **1996**, *87*, 1117–1157.

[29]	M. Stahn, S. Ehlert, S. Grimme, “Extended Conductor-like Polarizable Continuum Solvation Model (CPCM-X) for Semiempirical Methods” *J. Phys. Chem. A* **2023**, *127*, 7036–7043.

[30]	F. Lipparini, B. Stamm, E. Cancès, Y. Maday, B. Mennucci, “Fast Domain Decomposition Algorithm for Continuum Solvation Models: Energy and First Derivatives” *J. Chem. Theory Comput.* **2013**, *9*, 3637–3648.

[31]	E. Cancès, Y. Maday, B. Stamm, “Domain decomposition for implicit solvation models” *J. Chem. Phys.* **2013**, *139*, 054111.

[32]	N. Fleck, T. Hett, J. Brode, A. Meyer, S. Richert, O. Schiemann, “C–C Cross-Coupling Reactions of Trityl Radicals: Spin Density Delocalization, Exchange Coupling, and a Spin Label” *J. Org. Chem.* **2019**, *84*, 3293–3303.

[33]	C. Elsässer, M. Brecht, R. Bittl, “Pulsed Electron−Electron Double Resonance on Multinuclear Metal Clusters:  Assignment of Spin Projection Factors Based on the Dipolar Interaction” *J. Am. Chem. Soc.* **2002**, *124*, 12606–12611.
